# Supplementary material for: Transport-based transfer learning on Electronic Health Records: application to detection of treatment disparities
Source: J Am Med Inform Assoc. 2025 Sep 3;33(1):15–25. doi: 10.1093/jamia/ocaf134 (PMC12758479; doi:10.1093/jamia/ocaf134)
Supplement: ocaf134_Supplementary_Data [file ocaf134_supplementary_data.zip › JAMIA_OTTEHR_SI.pdf]

Supplemental Information for
Transport-based transfer learning on Electronic Health Records: Application to detection of treatment disparities

The Supplemental Information contains:

- Experiment details (Section 1), supplementary proof (Section 2) and descriptions on transfer learning methods (Section 3)
- Supplementary Figures S1–S6
- Supplementary Tables S1–S5

1 EXPERIMENT DETAILS

1.1 Data preprocessing

For MIMIC-III/MIMIC-IV, we first merged the admission table with the patient table by indexing unique patient IDs. For each admission, we calculated the duration in the hospital by taking the difference between the discharge time and the admission time (in seconds). For the cross-database experiment, we append each admission with MIMIC version (i.e., MIMIC-III or MIMIC-IV). For age experiments, we calculated the age of the patient in each admission by taking the difference between the admission time and date of birth.

1.2 Evaluation metrics

The evaluation metrics we used are *mean absolute error (MAE)* and *root mean squared error (RMSE)*. *MAE* measures the average magnitude of errors between predicted and actual values, providing an intuitive sense of the typical prediction error in the same units as the target variable, which gives by

$$\text{MAE} = \frac{1}{n}\sum_{i=1}^{n}\left|y_i - \hat{y}_i\right|,$$

where y_i is the actual value, $\hat{y}_i$ is the predicted value and n is the number of points. *RMSE* quantifies the standard deviation of prediction errors, emphasizing larger errors by squaring them, making it particularly sensitive to outliers.

$$\text{RMSE} = \sqrt{\frac{1}{n}\sum_{i=1}^{n}\left(y_i - \hat{y}_i\right)^2}.$$

2 PROOF OF THEOREM - UPPER BOUND FOR BINARY CLASSIFICATION AND REGRESSION

Let μ_T (μ_S) be a discrete target (source) distribution defined on a domain $D_T(D_S)$ and with probability mass function $\phi_T(\phi_S)$. Let $h' = h^* \circ T$, where h^* is Lipschitz continuous and T is the barycentric projection (Equation 4 in the main manuscript). The target error $\epsilon_T(h')$ defined by Equation 5 in the main manuscript is bounded by

$$\begin{aligned}
&\epsilon_T(h') \leq \epsilon_T(h^*, f_S) \\
&+ K\{\mathcal{W}_1(\mu_T, \mu_S) + M \sum_{x \in D_T} | \sum_{y \in D_S} \pi^*(x,y) - \phi_T(x)|\} \\
&+ \mathbb{E}_{x \sim \mu_T}\{|f_T(x) - f_S(x)|\},
\end{aligned}$$

where K is the Lipschitz continuous constant for h^*, π^* is the OT plan and $M = \max_{x \in D_T, y \in D_S} \|y - x\|$.

Proof. We first rewrite the target error as

$$\begin{aligned}
&\epsilon_T(h^* \circ T) = \epsilon_T(h^* \circ T, f_T) \\
&= \epsilon_T(h^* \circ T, f_T) + \epsilon_T(h^*, f_T) - \epsilon_T(h^*, f_T) \\
&+ \epsilon_T(h^*, f_S) - \epsilon_T(h^*, f_S) \\
&= \epsilon_T(h^*, f_S) + \underbrace{\epsilon_T(h^* \circ T, f_T) - \epsilon_T(h^*, f_T)}_{(*)} \\
&+ \underbrace{\epsilon_T(h^*, f_T) - \epsilon_T(h^*, f_S)}_{(**)}.
\end{aligned} \tag{1}$$

Since K is the Lipschitz constant for h^*, for all x, there exists $K > 0$ such that $\|h^* \circ T(x) - h^*(x)\| \leq K\|T(x) - x\|$. We now separately analyze $(*)$ and $(**)$. By the triangle inequality,

$$\begin{aligned}
&\epsilon_T(h^* \circ T, f_T) - \epsilon_T(h^*, f_T) \\
&= \mathop{\mathbb{E}}_{x \sim \mu_T}[\|h^* \circ T(x) - f_T(x)\|] - \mathop{\mathbb{E}}_{x \sim \mu_T}[\|h^*(x) - f_T(x)\|] \\
&= \mathop{\mathbb{E}}_{x \sim \mu_T}[\|h^* \circ T(x) - f_T(x)\| - \|h^*(x) - f_T(x)\|] \\
&\leq \mathop{\mathbb{E}}_{x \sim \mu_T}[\|h^* \circ T(x) - h^*(x)\|] \\
&\leq \mathop{\mathbb{E}}_{x \sim \mu_T}[K\|T(x) - x\|] \\
&= K \mathop{\mathbb{E}}_{x \sim \mu_T}[\|T(x) - x\|],
\end{aligned} \tag{2}$$

Let $\rho(x) = \frac{\phi_T(x)}{\sum_{y \in D_S} \pi^*(x,y)}$ ($\rho(x)$ is well defined since $\sum_{y \in D_S} \pi^*(x,y) \neq 0$). We then obtain

$$\begin{aligned}
&\mathop{\mathbb{E}}_{x \sim \mu_T}[\|T(x) - x\|] \\
&= \sum_{x \in D_T} \|T(x) - x\| \phi_T(x) \\
&= \sum_{x \in D_T} (\|[\frac{1}{\sum_{y \in D_S} \pi^*(x,y)} \sum_{y \in D_S} y\pi^*(x,y)] - x\| \rho(x) \sum_{y \in D_S} \pi^*(x,y)) \\
&= \sum_{x \in D_T} \|\rho(x) \sum_{y \in D_S} y\pi^*(x,y) - \rho(x) \sum_{y \in D_S} \pi^*(x,y)x\| \\
&= \sum_{x \in D_T} \rho(x) \sum_{y \in D_S} \|(y - x)\pi^*(x,y)\| \\
&= \sum_{x \in D_T} (1 - (1 - \rho(x))) \sum_{y \in D_S} \|(y - x)\pi^*(x,y)\|.
\end{aligned} \tag{3}$$

We can thus further bound $\mathbb{E}_{x\sim\mu_T}[\|T(x)-x\|]$ as

$$\begin{aligned}
&\mathop{\mathbb{E}}_{x\sim\mu_T}[\|T(x)-x\|] \le \sum_{x\in D_T}\sum_{y\in D_S}\|y-x\|\pi^*(x,y)\\
&+\sum_{x\in D_T}|1-\rho(x)|\sum_{y\in D_S}\|y-x\|\pi^*(x,y)\\
&\le \sum_{x\in D_T}\sum_{y\in D_S}\|y-x\|\pi^*(x,y)-\frac{1}{\lambda}\mathcal{H}(\pi^*)\\
&+D_\varphi(\pi_S^*|\phi_S)+D_\varphi(\pi_T^*|\phi_T)\\
&+\sum_{x\in D_T}|1-\rho(x)|\sum_{y\in D_S}\|y-x\|\pi^*(x,y)\\
&\le \mathcal{W}(\mu_T,\mu_S)+M\sum_{x\in D_T}|1-\rho(x)|\sum_{y\in D_S}\pi^*(x,y),
\end{aligned} \tag{4}$$

where $M=\max_{x\in D_T,y\in D_S}\|y-x\|$. Finally,

$$\begin{aligned}
&\mathop{\mathbb{E}}_{x\sim\mu_T}[\|T(x)-x\|]\\
&\le \mathcal{W}(\mu_T,\mu_S)+M\sum_{x\in D_T}|1-\frac{\phi_T(x)}{\sum_{y\in D_S}\pi^*(x,y)}|\sum_{y\in D_S}\pi^*(x,y)\\
&= \mathcal{W}(\mu_T,\mu_S)+M\sum_{x\in D_T}|\sum_{y\in D_S}\pi^*(x,y)-\phi_T(x)|,
\end{aligned} \tag{5}$$

which yields the upper bound for $(*)$

$$\begin{aligned}
&\epsilon_T(h^*\circ T,f_T)-\epsilon_T(h^*,f_T)\\
&\le K\{\mathcal{W}(\mu_T,\mu_S)+M\sum_{x\in D_T}|\sum_{y\in D_S}\pi^*(x,y)-\phi_T(x)|\}.
\end{aligned} \tag{6}$$

Considering $(**)$, we have by triangle inequality,

$$\begin{aligned}
&\epsilon_T(h^*,f_T)-\epsilon_T(h^*,f_S)\\
&=\mathop{\mathbb{E}}_{x\sim\mu_S}[|h^*(x)-f_T(x)|]-\mathop{\mathbb{E}}_{x\sim\mu_S}[|h^*(x)-f_S(x)|]\\
&\le \mathop{\mathbb{E}}_{x\sim\mu_S}[|f_T(x)-f_S(x)|].
\end{aligned} \tag{7}$$

Plugging Equations (6) and (7) into Equation (1) yields

$$\begin{aligned}
&\epsilon_T(h^*\circ T)\le \epsilon_T(h^*,f_S)\\
&+K\{\mathcal{W}(\mu_T,\mu_S)+M\sum_{x\in D_T}|\sum_{y\in D_S}\pi^*(x,y)-\phi_T(x)|\}\\
&+\mathop{\mathbb{E}}_{x\sim\mu_T}[|f_T(x)-f_S(x)|],
\end{aligned} \tag{8}$$

which completes the proof.

We note that when h^* is linear, Equation (2) can be rewritten in the following way:

$$\begin{aligned}
&\epsilon_T(h^*\circ T,f_T)-\epsilon_T(h^*,f_T)\\
&\le \mathop{\mathbb{E}}_{x\sim\mu_T}[\|h^*\circ T(x)-h^*(x)\|]\\
&= \mathop{\mathbb{E}}_{x\sim\mu_T}[\|h^*(T(x)-(x)\|]\\
&\le \mathop{\mathbb{E}}_{x\sim\mu_T}[\|\|h^*\|\|\|T(x)-x\|]\\
&= \|\|h^*\|\|\mathop{\mathbb{E}}_{x\sim\mu_T}[\|T(x)-x\|],
\end{aligned} \tag{9}$$

where $\|\|h^*\|\| = \underset{\|x\|=1, x\in D_T}{sup} \|h^*(x)\|$.

In this case, the theorem can be rewritten as:

$$\begin{aligned} \underbrace{\epsilon_T(h')}_{\text{target error}} \leq & \underbrace{\epsilon_T(h^*, f_S)}_{\text{source model error on the target domain}} \\ & + \underbrace{\|\|h^*\|\|\{\mathcal{W}_1(\mu_T, \mu_S) + M \sum_{x\in D_T} | \sum_{y\in D_S} \pi^*(x,y) - \phi_T(x)|\}}_{\text{transport}} \\ & + \underbrace{\mathbb{E}_{x\sim\mu_T}\{|f_T(x) - f_S(x)|\}}_{\text{labeling divergence}}, \end{aligned}$$

where $\|\|h^*\|\| = \underset{\|x\|=1, x\in D_T}{sup} \|h^*(x)\|$.

□

3 OVERVIEW OF METHODS FOR TRANSFER LEARNING AND METHODS USED IN BENCHMARKING

We provide here a short overview of standard methods used for Transfer Learning, with a focus on standard statistical, Machine Learning OT and non-OT -based methods. Standard statistical methods such as Correlation Alignment (*CA*)[1], Transfer Component Analysis (*TCA*)[2], Euclidean space data alignment[3] and Geodesic Flow Kernel (*GFK*)[4] tackle TL by aligning statistical properties between source and target domains, supporting both classification and regression tasks. In machine learning, OT-based TL has been, to the best of our knowledge, exclusively been studied for classification tasks and more specifically tested on image classification tasks, with methods such as Joint Distribution Adaptation[5], Deep Joint Distribution Optimal Transport (*deepJDOT*)[6], and Class-aware Sample Reweighting[7]. These methods leverage OT to reduce the Wasserstein distance between source and target domains, thereby aligning their distributions. On the other hand, non-OT-based TL has been studied for classification including moment-matching methods[8–10] and adversarial learning methods [11–17] as well as for regression such as Representation Subspace Distance (*RSD*)[18] and inverse GRAM matrices (*daregram*)[19], which learn a shared feature extractor by minimizing some discrepancies of source and target features.

4 SUPPLEMENTARY FIGURES

4.1 Feature embedding differences

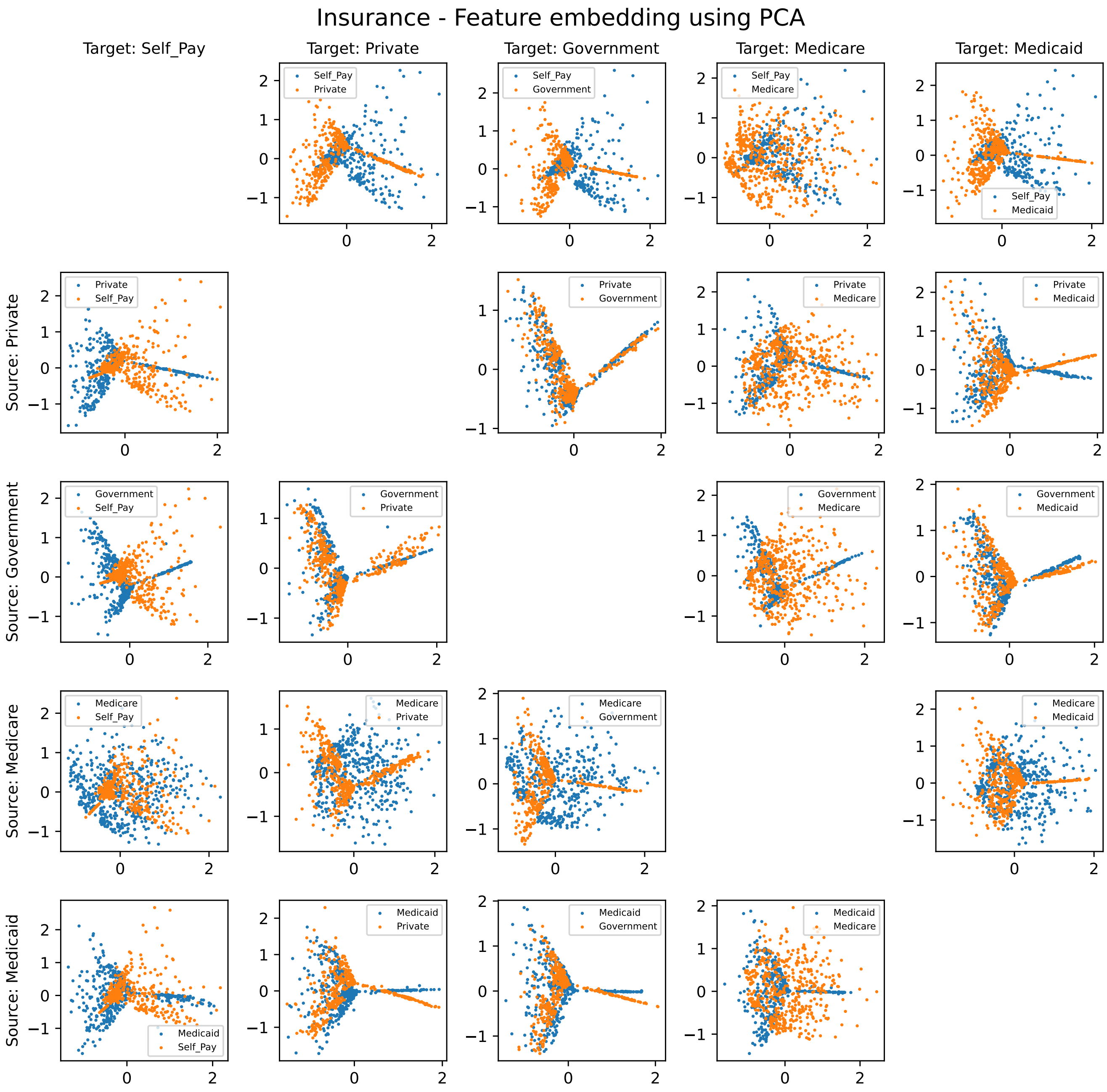

Figure S1. Feature embedding for pairwise insurance groups using PCA in the dimension of the first two principal components. Insurance groups include “Self_Pay,” “Private,” “Government,” “Medicare,” and “Medicaid.”

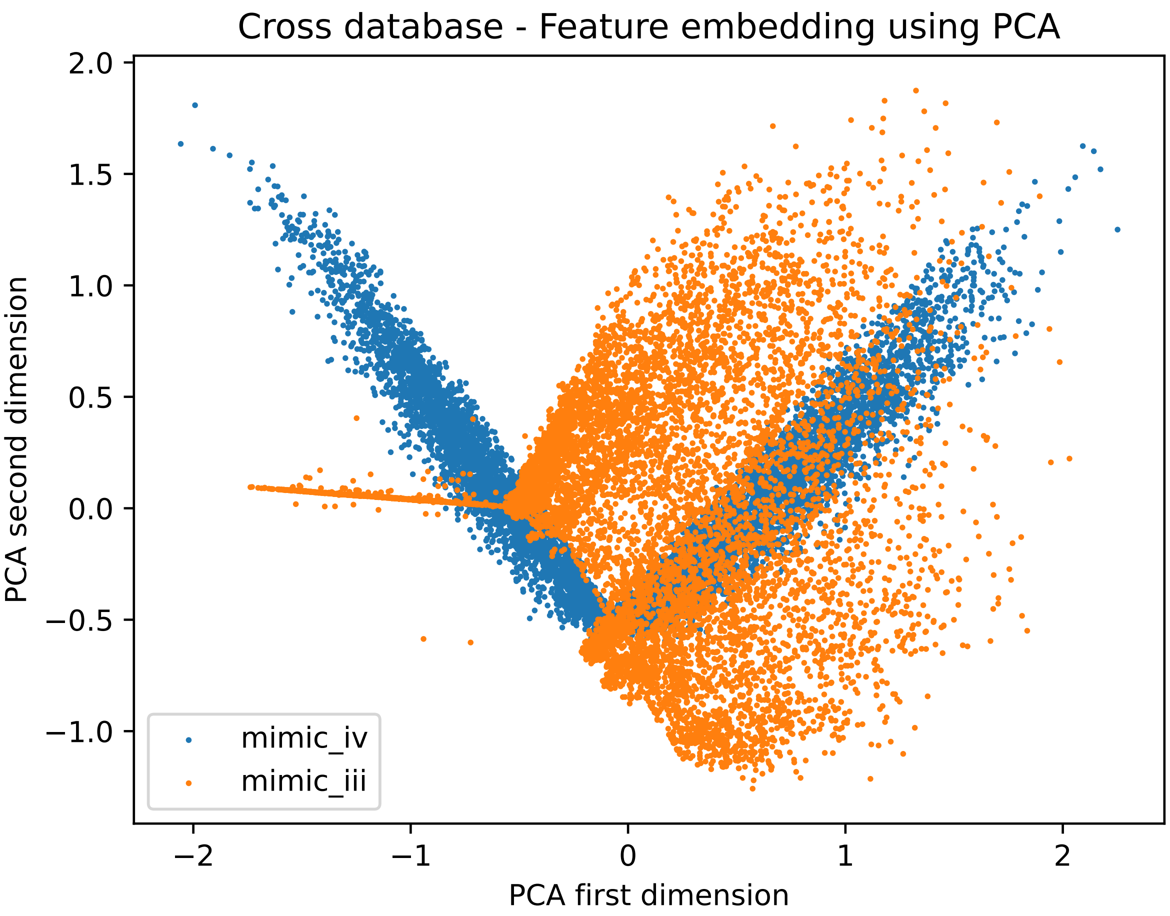

Figure S2. Feature embedding for MIMIC-IV (source) and MIMIC-III (target) using PCA in the dimension of the first two principal components.

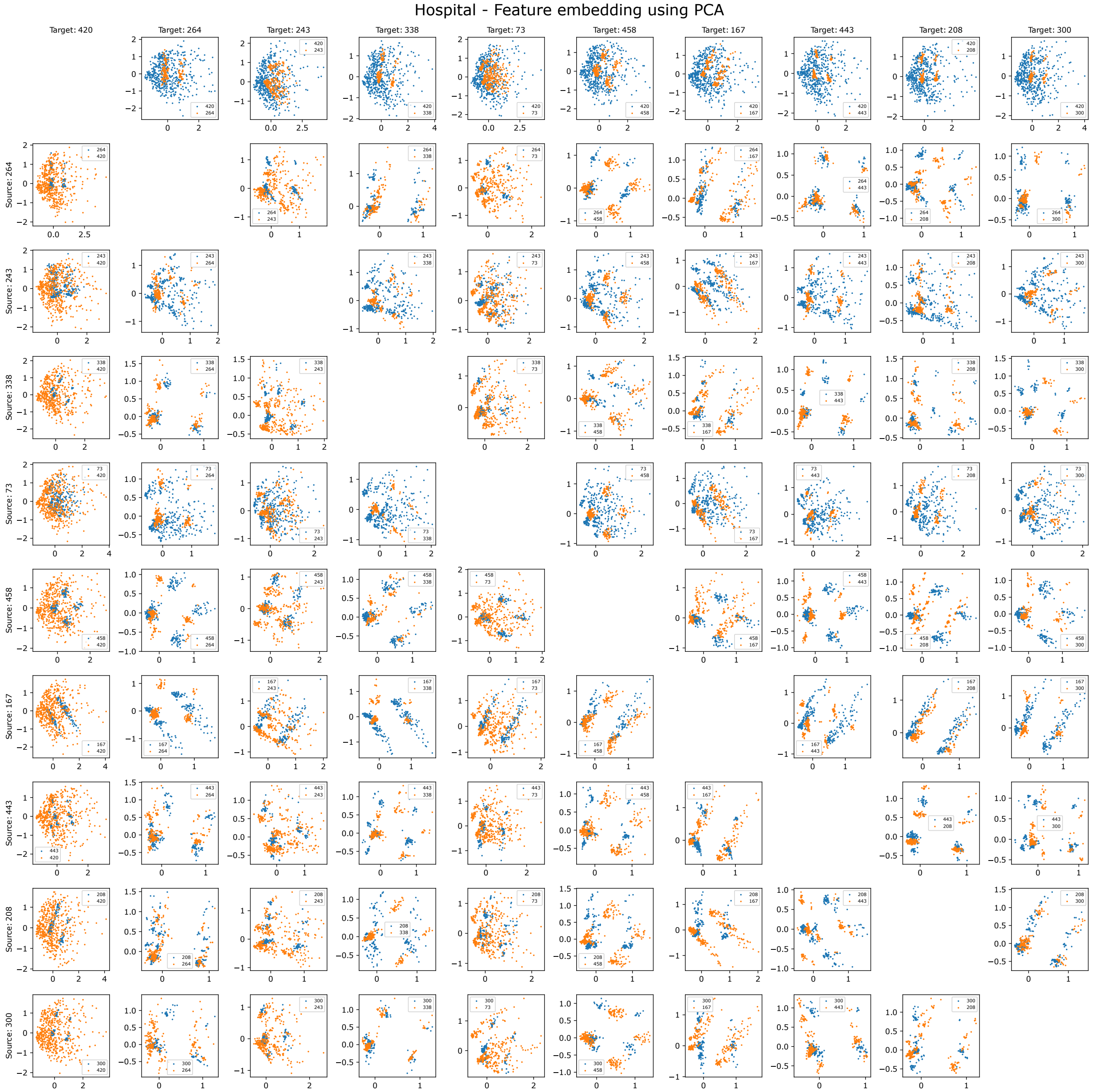

Figure S3. Feature embedding for pairwise hospital groups using PCA in the dimension of the first two principal components. Hospital IDs include 420, 264, 243, 338, 73, 458, 167, 443, 208 and 300.

4.2 Illustration of $\epsilon_T(h^*, f_S)$ in Theorem 1

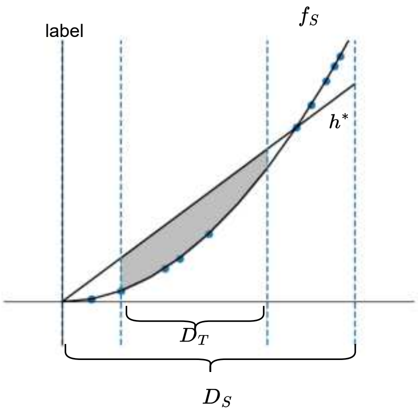

Figure S4. Illustration of $\epsilon_T(h^*, f_S)$. D_S and D_T are source and target embedding spaces. The blue dots denote the source embeddings and source labels. f_S is the ground-truth labeling function for source embedding features and source labels. h^* is the source model trained by source embedding features and source labels. The gray area denotes $\epsilon_T(h^*, f_S)$.

4.3 Bound analysis

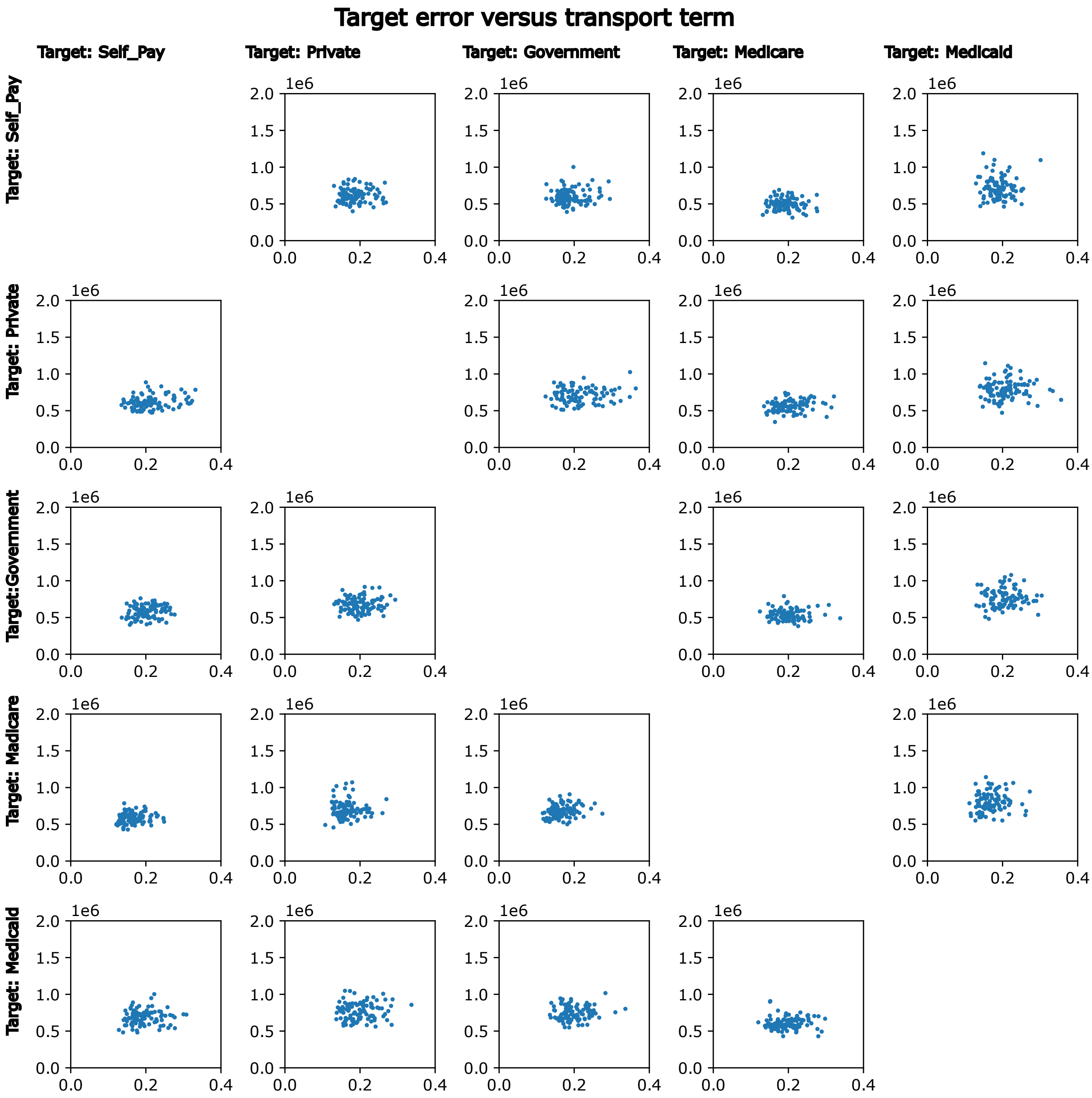

Figure S5. Bound analysis for pairwise insurance group experiments with respect to transport term. Target error versus transport term for pairwise insurance groups with an average PCC of 0.09.

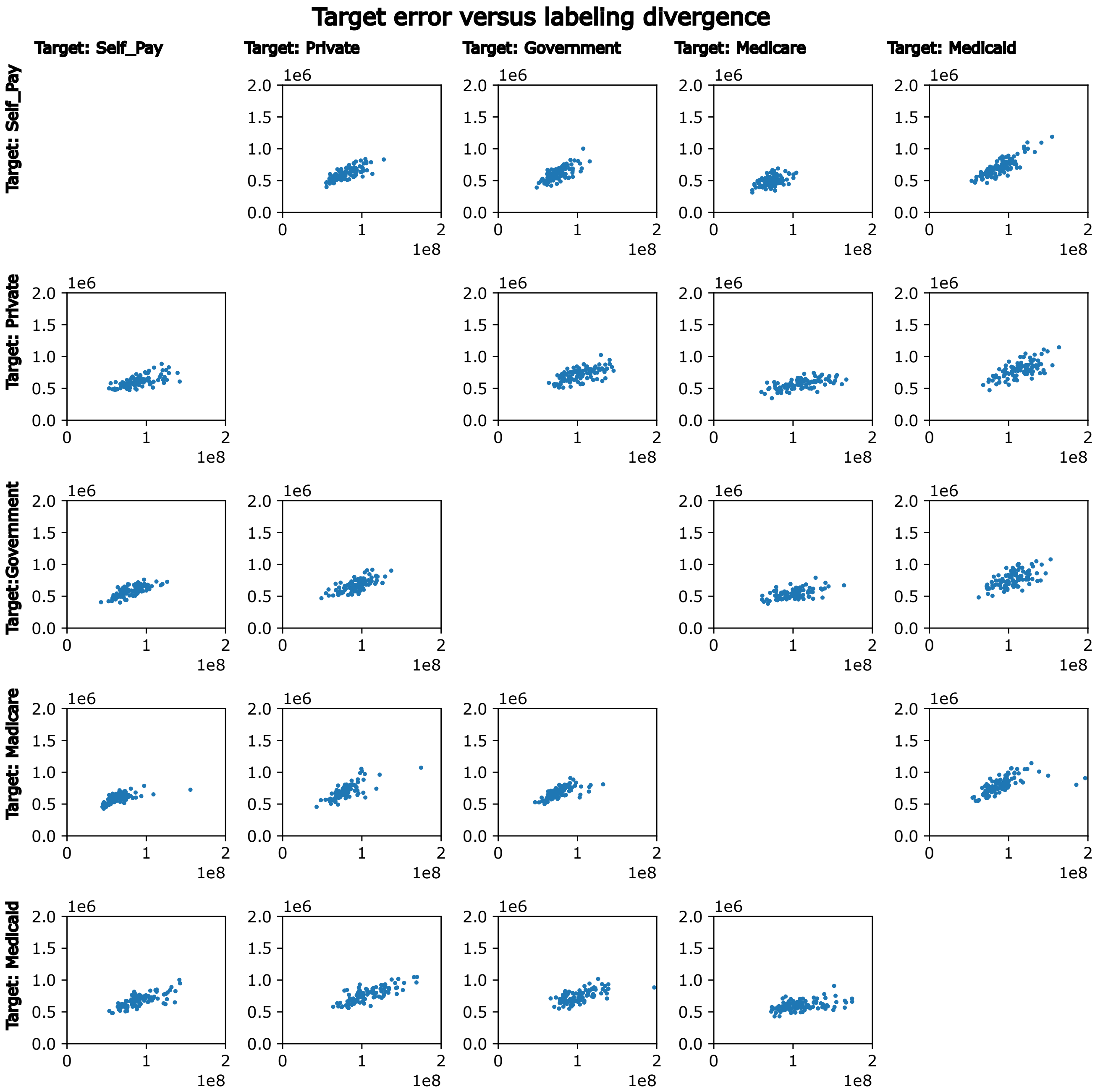

Figure S6. Bound analysis for pairwise insurance group experiments with respect to labeling divergence. Target error versus labeling divergence for pairwise insurance groups with an average PCC of 0.67.

4.4 Admissions with significantly reduced durations in hospital for all pairwase experiments

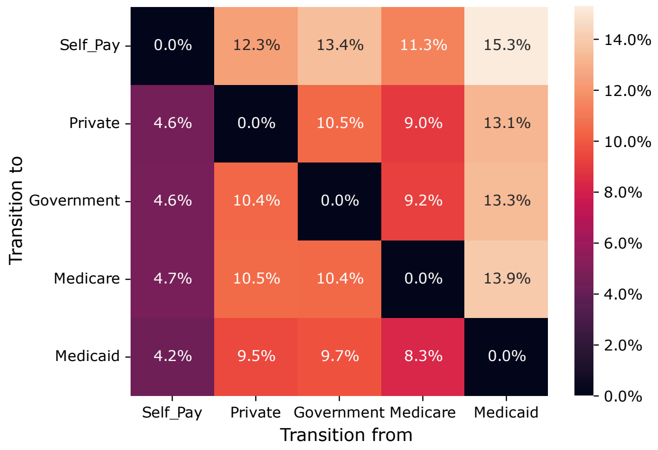

Figure S7. Percentages of admissions having significantly reduced durations in hospital for all pairwise insurance group experiments. For example, when transitioning to self paid insurance plans, 15.3% of the admissions on Medicaid would result in significantly reduced durations in hospital.

5 SUPPLEMENTARY TABLES

5.1 Mann-Whitney U test result for different experiments

Table S1. P-values from Mann-Whitney U tests to test the difference in distribution medians for the first two dimensions in PCA embeddings between pairwise insurance groups for insurance experiments. The significant p-values (< 0.05) are highlighted in red.

Source	Target	dimension 1	dimension 2
Self_Pay	Private	2.08e-02	3.74e-02
Self_Pay	Government	1.20e-01	5.41e-01
Self_Pay	Medicare	9.45e-02	3.74e-01
Self_Pay	Medicaid	1.79e-01	1.45e-01
Private	Self_Pay	2.03e-01	2.87e-03
Private	Government	4.81e-01	6.91e-01
Private	Medicare	1.16e-01	4.05e-01
Private	Medicaid	6.78e-01	9.21e-01
Government	Self_Pay	3.77e-01	1.54e-01
Government	Private	6.72e-01	2.91e-01
Government	Medicare	5.37e-03	6.38e-01
Government	Medicaid	8.21e-01	4.19e-01
Medicare	Self_Pay	6.89e-01	1.98e-01
Medicare	Private	8.58e-01	1.64e-01
Medicare	Government	2.88e-01	9.17e-01
Medicare	Medicaid	7.73e-01	6.56e-01
Medicaid	Self_Pay	1.46e-01	2.82e-02
Medicaid	Private	5.71e-02	4.99e-01
Medicaid	Government	3.10e-01	8.42e-01
Medicaid	Medicare	1.92e-02	2.96e-01

Table S2. P-values from Mann-Whitney U tests to test the difference in distribution medians for the first two dimensions in PCA embeddings between pairwise insurance groups for cross-database experiments. The significant p-values (< 0.05) are highlighted in red.

Source	Target	dimension 1	dimension 2
MIMIC-IV	MIMIC-III	5.39e-01	9.44e-08

Table S3. P-values from Mann-Whitney U tests to test the difference in distribution medians for the first two dimensions in PCA embeddings between pairwise hospitals for cross-hospital experiments. The significant p-values (<0.05) are highlighted in red.

Source	Target	Dimension 1	Dimension 2
420	264	1.86e-02	1.87e-02
420	243	2.06e-02	7.46e-01
420	338	1.54e-03	6.63e-02
420	73	1.13e-01	5.40e-01
420	458	4.80e-02	6.11e-01
420	167	2.16e-02	1.88e-01
420	443	2.23e-04	2.97e-01
420	208	2.06e-03	6.32e-01
420	300	2.27e-04	1.23e-01
264	420	1.47e-03	1.09e-01
264	243	1.24e-05	1.51e-03
264	338	4.91e-06	2.08e-03
264	73	5.04e-05	4.79e-10
264	458	1.06e-15	2.61e-15
264	167	6.50e-12	2.32e-02
264	443	1.49e-01	2.41e-08
264	208	1.23e-10	3.96e-03
264	300	4.02e-01	8.48e-23
243	420	7.98e-03	8.67e-01
243	264	1.09e-04	2.42e-01
243	338	1.48e-03	3.93e-12
243	73	3.88e-01	5.64e-01
243	458	1.48e-03	2.31e-09
243	167	5.80e-02	8.58e-02
243	443	5.84e-04	8.12e-02
243	208	1.34e-01	1.54e-10
243	300	7.24e-11	8.64e-08
338	420	2.72e-04	9.26e-01
338	264	1.10e-10	1.44e-07
338	243	3.84e-05	7.09e-02
338	73	2.38e-05	6.55e-06
338	458	2.92e-13	6.63e-14
338	167	5.61e-13	1.54e-16
338	443	3.36e-02	2.03e-02
338	208	4.51e-20	9.30e-01
338	300	4.44e-06	5.64e-13
73	420	5.28e-02	5.25e-01
73	264	8.80e-05	4.75e-03
73	243	1.48e-01	4.99e-01
73	338	3.55e-04	1.59e-02
73	458	1.71e-03	6.37e-02
73	167	6.12e-03	1.38e-03
73	443	2.84e-03	9.99e-06
73	208	8.45e-03	7.82e-02
73	300	1.10e-05	3.70e-08
458	243	1.48e-03	4.06e-02
458	338	8.57e-19	1.04e-07
458	73	3.40e-02	4.80e-03
458	167	1.36e-07	5.00e-06
458	443	1.36e-14	4.05e-14
458	208	4.62e-11	5.51e-24
458	300	7.81e-20	7.75e-03
167	420	2.06e-02	1.42e-01
167	264	4.18e-12	1.59e-06
167	243	7.08e-01	2.09e-04
167	338	3.75e-14	4.52e-13
167	73	1.75e-01	1.33e-01
167	458	4.11e-05	1.65e-01
167	443	5.51e-13	6.42e-05
167	208	7.90e-18	4.13e-05
167	300	3.88e-15	1.64e-04
443	420	4.04e-03	5.46e-01
443	264	3.08e-01	7.18e-10
443	243	6.07e-04	6.30e-03
443	338	5.02e-18	6.87e-14
443	73	1.27e-05	1.41e-03
443	458	3.35e-13	5.57e-02
443	167	3.14e-12	3.07e-23
443	208	3.11e-05	8.78e-02
443	300	4.97e-09	3.99e-40
208	420	1.04e-02	2.39e-01
208	264	3.00e-09	7.67e-08
208	243	3.33e-08	1.20e-03
208	338	4.54e-08	3.52e-20
208	73	4.04e-04	4.14e-03
208	458	3.65e-11	5.54e-10
208	167	2.71e-12	2.24e-12
208	443	7.28e-01	3.54e-09
208	300	4.98e-26	3.69e-18
300	420	1.75e-03	3.32e-01
300	264	1.72e-15	6.31e-01
300	243	2.68e-05	3.22e-14
300	338	1.91e-02	2.60e-07
300	73	1.94e-04	5.59e-05
300	458	9.68e-20	9.01e-05
300	167	4.82e-16	4.25e-01
300	443	3.96e-17	1.52e-11
300	208	7.16e-15	4.63e-14

5.2 Benchmark results for pairwise insurance group experiments

Table S4. Benchmark results for pairwise insurance group experiments. Medians and standard deviations of the log of *MAE* between projected duration and observed duration on target admissions for different insurance groups using *OTTEHR*, *TCA*, *CA*, *GFK*, *deepJDOT*, *RSD* and *daregram*. The outperformance ratio of *OTTEHR* to *TCA*/*CA*/*GFK*/*RSD*/*daregram* is defined as the percentage decrease in the median *MAE* from *TCA*/*CA*/*GFK*/*RSD*/*daregram* to *OTTEHR*. The outperformance ratio of *OTTEHR* to *deepJDOT* is defined as the percentage decrease in median of the log-transformed of standard deviation of *MAE* from *deepJDOT* to *OTTEHR*.

Source	Target	*OTTEHR*	*TCA*	*CA*	*GFK*	*deepJDOT*	*RSD*	*daregram*
Self_Pay	Private	13.40(0.16)	13.60(0.17)	13.63(0.18)	13.60(0.17)	13.32(0.18)	13.68(0.13)	13.66(0.14)
Self_Pay	Government	13.35(0.15)	13.55(0.16)	13.56(0.17)	13.56(0.16)	13.32(0.17)	13.64(0.12)	13.62(0.13)
Self_Pay	Medicare	13.28(0.14)	13.54(0.14)	13.58(0.15)	13.55(0.15)	13.13(0.47)	13.64(0.11)	13.67(0.10)
Self_Pay	Medicaid	13.53(0.18)	13.74(0.18)	13.73(0.18)	13.73(0.18)	13.46(0.29)	13.79(0.14)	13.78(0.12)
Private	Self_Pay	13.36(0.17)	13.78(0.19)	13.80(0.17)	13.78(0.20)	13.30(0.87)	13.21(0.13)	13.23(0.10)
Private	Government	13.52(0.16)	13.83(0.18)	13.86(0.19)	13.84(0.18)	13.44(0.31)	13.62(0.14)	13.61(0.11)
Private	Medicare	13.48(0.16)	13.95(0.21)	13.94(0.21)	13.94(0.21)	13.19(0.13)	13.63(0.10)	13.66(0.10)
Private	Medicaid	13.65(0.18)	13.98(0.17)	13.99(0.17)	13.98(0.17)	13.55(0.77)	13.82(0.13)	13.81(0.14)
Government	Self_Pay	13.28(0.14)	13.66(0.18)	13.69(0.18)	13.67(0.18)	13.25(0.12)	13.22(0.09)	13.25(0.12)
Government	Private	13.45(0.16)	13.78(0.17)	13.82(0.18)	13.78(0.17)	13.49(1.01)	13.66(0.14)	13.67(0.12)
Government	Medicare	13.42(0.15)	13.85(0.19)	13.84(0.19)	13.85(0.19)	13.17(0.80)	13.66(0.10)	13.66(0.09)
Government	Medicaid	13.62(0.16)	13.91(0.16)	13.93(0.17)	13.92(0.16)	13.56(0.55)	13.81(0.13)	13.82(0.14)
Medicare	Self_Pay	13.23(0.13)	13.44(0.18)	13.47(0.18)	13.44(0.18)	13.28(0.81)	13.22(0.12)	13.22(0.12)
Medicare	Private	13.44(0.17)	13.62(0.17)	13.65(0.18)	13.62(0.17)	13.45(1.06)	13.67(0.14)	13.68(0.12)
Medicare	Government	13.42(0.13)	13.58(0.17)	13.62(0.15)	13.57(0.17)	13.43(0.36)	13.64(0.13)	13.64(0.12)
Medicare	Medicaid	13.61(0.17)	13.74(0.20)	13.78(0.20)	13.74(0.20)	13.55(0.22)	13.81(0.11)	13.77(0.14)
Medicaid	Self_Pay	13.42(0.18)	13.77(0.19)	13.81(0.20)	13.78(0.19)	13.42(0.87)	13.23(0.12)	13.22(0.11)
Medicaid	Private	13.64(0.18)	13.88(0.20)	13.95(0.19)	13.87(0.20)	13.54(0.17)	13.66(0.16)	13.66(0.13)
Medicaid	Government	13.55(0.16)	13.87(0.17)	13.89(0.16)	13.87(0.18)	13.53(0.81)	13.63(0.13)	13.66(0.13)
Medicaid	Medicare	13.54(0.16)	13.92(0.21)	13.96(0.21)	13.92(0.21)	13.32(0.25)	13.66(0.10)	13.65(0.10)
Population Median		13.46(0.20)	13.75(0.23)	13.78(0.23)	13.75(0.23)	13.39(0.62)	13.64(0.24)	13.64(0.23)
Outperformance ratio of *OTTEHR*		N.A.	25.17	27.39	25.17	67.74	16.47	16.47

Table S5. Benchmark results for pairwise insurance group experiments. Medians and standard deviations of the log of *RMSE* between projected duration and observed duration on target admissions for different insurance groups using *OTTEHR*, *TCA*, *CA*, *GFK*, *deepJDOT*, *RSD* and *daregram*. The outperformance ratio of *OTTEHR* to *TCA*/*CA*/*GFK*/*RSD*/*daregram* is defined as the percentage decrease in the median *RMSE* from *TCA*/*CA*/*GFK*/*RSD*/*daregram* to *OTTEHR*. The outperformance ratio of *OTTEHR* to *deepJDOT* is defined as the percentage decrease in median of the log-transformed of standard deviation of *RMSE* from *deepJDOT* to *OTTEHR*.

Source	Target	*OTTEHR*	*TCA*	*CA*	*GFK*	*deepJDOT*	*RSD*	*daregram*
Self_Pay	Private	14.03(0.23)	14.11(0.22)	14.15(0.23)	14.09(0.22)	13.99(0.25)	14.19(0.19)	14.17(0.21)
Self_Pay	Government	13.95(0.20)	14.02(0.19)	14.03(0.19)	14.03(0.19)	13.90(0.23)	14.11(0.17)	14.11(0.18)
Self_Pay	Medicare	13.71(0.20)	13.90(0.17)	13.89(0.17)	13.89(0.17)	13.64(0.45)	13.93(0.19)	13.97(0.16)
Self_Pay	Medicaid	14.15(0.25)	14.24(0.23)	14.24(0.22)	14.24(0.23)	14.16(0.30)	14.30(0.20)	14.29(0.17)
Private	Self_Pay	13.85(0.23)	14.08(0.21)	14.05(0.18)	14.07(0.21)	13.53(0.86)	13.63(0.19)	13.69(0.16)
Private	Government	14.05(0.23)	14.22(0.20)	14.22(0.21)	14.23(0.19)	13.82(0.31)	14.09(0.20)	14.08(0.17)
Private	Medicare	13.95(0.21)	14.27(0.21)	14.24(0.21)	14.25(0.21)	13.53(0.22)	13.95(0.14)	13.97(0.14)
Private	Medicaid	14.24(0.24)	14.38(0.20)	14.37(0.20)	14.37(0.19)	14.15(0.74)	14.33(0.19)	14.32(0.21)
Government	Self_Pay	13.72(0.22)	13.94(0.19)	13.97(0.18)	13.96(0.20)	13.49(0.17)	13.67(0.16)	13.66(0.17)
Government	Private	14.05(0.23)	14.18(0.20)	14.24(0.21)	14.19(0.19)	14.01(0.96)	14.15(0.21)	14.19(0.19)
Government	Medicare	13.88(0.22)	14.16(0.19)	14.16(0.19)	14.16(0.19)	13.58(0.78)	13.97(0.15)	13.96(0.18)
Government	Medicaid	14.18(0.21)	14.28(0.18)	14.33(0.18)	14.30(0.18)	14.13(0.54)	14.33(0.21)	14.35(0.20)
Medicare	Self_Pay	13.56(0.18)	13.71(0.20)	13.71(0.19)	13.70(0.20)	13.49(0.79)	13.67(0.19)	13.68(0.19)
Medicare	Private	13.98(0.24)	14.06(0.21)	14.09(0.22)	14.05(0.21)	13.97(0.99)	14.18(0.22)	14.17(0.19)
Medicare	Government	13.88(0.20)	13.96(0.20)	14.01(0.18)	13.96(0.20)	13.91(0.36)	14.10(0.17)	14.08(0.18)
Medicare	Medicaid	14.18(0.24)	14.21(0.24)	14.25(0.24)	14.22(0.24)	14.12(0.29)	14.33(0.17)	14.29(0.21)
Medicaid	Self_Pay	13.84(0.22)	14.07(0.19)	14.07(0.20)	14.06(0.19)	13.60(0.86)	13.71(0.17)	13.66(0.18)
Medicaid	Private	14.15(0.23)	14.26(0.21)	14.29(0.20)	14.25(0.21)	14.01(0.24)	14.18(0.23)	14.14(0.19)
Medicaid	Government	14.02(0.20)	14.18(0.18)	14.22(0.16)	14.18(0.18)	13.89(0.78)	14.10(0.18)	14.14(0.18)
Medicaid	Medicare	13.97(0.23)	14.22(0.21)	14.23(0.22)	14.21(0.21)	13.65(0.28)	13.95(0.18)	13.97(0.16)
Population Median		13.96(0.28)	14.12(0.25)	14.14(0.25)	14.12(0.25)	13.80(0.63)	14.05(0.29)	14.05(0.28)
Outperformance ratio of *OTTEHR*		N.A.	14.79	16.47	14.79	55.56	8.61	8.61

Table S6. Benchmark results for cross-database experiments. Medians and standard deviations of the log of *MAE* between projected duration and observed duration on target admissions for the cross-database experiment using *OTTEHR*, *TCA*, *CA*, *GFK*, *deepJDOT*, *RSD* and *daregram*. The outperformance ratio of *OTTEHR* to *TCA*/*CA*/*GFK*/*RSD*/*daregram* is defined as the percentage decrease in the median *MAE* from *TCA*/*CA*/*GFK*/*RSD*/*daregram* to *OTTEHR*. The outperformance ratio of *OTTEHR* to *deepJDOT* is defined as the percentage decrease in median of the log-transformed of standard deviation of *MAE* from *deepJDOT* to *OTTEHR*.

Source	Target	*OTTEHR*	*TCA*	*CA*	*GFK*	*deepJDOT*	*RSD*	*daregram*
MIMIC-IV	MIMIC-III	13.25(0.18)	13.26(0.18)	13.49(0.16)	13.50(0.17)	13.25(0.18)	13.68(0.12)	13.67(0.13)
Outperformance ratio of *OTTEHR*		N.A.	1.01	27.12	28.40	50.00	53.73	52.20

Table S7. Benchmark results for cross-database experiments. Medians and standard deviations of the log of *RMSE* between projected duration and observed duration on target admissions for the cross-database experiment using *OTTEHR*, *TCA*, *CA*, *GFK*, *deepJDOT*, *RSD* and *daregram*. The outperformance ratio of *OTTEHR* to *TCA*/*CA*/*GFK*/*RSD*/*daregram* is defined as the percentage decrease in the median *RMSE* from *TCA*/*CA*/*GFK*/*RSD*/*daregram* to *OTTEHR*. The outperformance ratio of *OTTEHR* to *deepJDOT* is defined as the percentage decrease in median of the log-transformed of standard deviation of *RMSE* from *deepJDOT* to *OTTEHR*.

Source	Target	*OTTEHR*	*TCA*	*CA*	*GFK*	*deepJDOT*	*RSD*	*daregram*
MIMIC-IV	MIMIC-III	13.90(0.30)	13.94(0.29)	14.01(0.24)	14.01(0.24)	13.90(0.41)	14.08(0.20)	14.05(0.23)
Outperformance ratio of *OTTEHR*		N.A.	3.92	11.17	11.17	36.67	18.95	15.55

Table S8. Benchmark results for pairwise cross-hospital experiments. Medians and standard deviations of the log of *MAE* between projected duration and observed duration on target admissions for different insurance groups using *OTTEHR*, *TCA*, *CA*, *GFK*, *deepJDOT*, *RSD* and *daregram*. The outperformance ratio of *OTTEHR* to *TCA*/*CA*/*GFK*/*RSD*/*daregram* is defined as the percentage decrease in the median *MAE* from *TCA*/*CA*/*GFK*/*RSD*/*daregram* to *OTTEHR*. The outperformance ratio of *OTTEHR* to *deepJDOT* is defined as the percentage decrease in median of the log-transformed of standard deviation of *MAE* from *deepJDOT* to *OTTEHR*.

Source	Target	*OTTEHR*	*TCA*	*CA*	*GFK*	*deepJDOT*	*RSD*	*daregram*
420	264	13.07(0.13)	13.20(0.12)	13.29(0.13)	13.27(0.12)	19.02(1.91)	13.41(0.08)	13.42(0.09)
420	243	13.08(0.15)	13.19(0.12)	13.38(0.15)	13.28(0.13)	18.80(1.92)	13.40(0.10)	13.42(0.11)
420	338	13.11(0.11)	13.22(0.10)	13.32(0.11)	13.28(0.10)	18.60(1.72)	13.49(0.09)	13.50(0.10)
420	73	13.37(0.16)	13.37(0.15)	13.52(0.16)	13.46(0.15)	18.64(1.76)	13.74(0.12)	13.76(0.11)
420	458	13.42(0.29)	13.48(0.27)	13.57(0.25)	13.52(0.26)	18.71(1.34)	13.76(0.21)	13.75(0.22)
420	167	13.29(0.14)	13.34(0.12)	13.47(0.15)	13.38(0.13)	18.43(1.33)	13.67(0.10)	13.64(0.12)
420	443	13.52(0.14)	13.53(0.13)	13.58(0.13)	13.58(0.13)	18.87(1.49)	13.87(0.12)	13.87(0.11)
420	208	13.30(0.16)	13.35(0.16)	13.45(0.16)	13.41(0.15)	18.80(1.97)	13.65(0.12)	13.67(0.15)
420	300	13.17(0.15)	13.29(0.13)	13.35(0.14)	13.33(0.14)	18.91(1.80)	13.54(0.11)	13.54(0.11)
264	420	13.38(0.13)	13.52(0.19)	13.99(0.19)	13.85(0.15)	13.64(1.99)	13.68(0.10)	13.69(0.11)
264	243	13.10(0.13)	13.13(0.16)	13.48(0.20)	13.34(0.16)	14.05(1.87)	13.42(0.11)	13.43(0.10)
264	338	13.13(0.12)	13.12(0.13)	13.35(0.17)	13.25(0.15)	14.23(1.37)	13.49(0.09)	13.49(0.08)
264	73	13.39(0.14)	13.40(0.16)	13.71(0.16)	13.58(0.15)	14.25(1.20)	13.76(0.11)	13.74(0.10)
264	458	13.43(0.27)	13.42(0.27)	13.65(0.25)	13.57(0.26)	14.15(2.10)	13.75(0.23)	13.70(0.18)
264	167	13.30(0.13)	13.33(0.13)	13.57(0.16)	13.46(0.13)	14.52(2.95)	13.65(0.11)	13.65(0.11)
264	443	13.55(0.14)	13.53(0.14)	13.70(0.15)	13.65(0.13)	14.15(1.73)	13.87(0.12)	13.87(0.12)
264	208	13.30(0.17)	13.29(0.17)	13.47(0.16)	13.42(0.16)	14.20(1.33)	13.63(0.14)	13.64(0.14)
264	300	13.21(0.14)	13.19(0.14)	13.43(0.15)	13.34(0.12)	14.12(2.02)	13.53(0.11)	13.54(0.10)
243	420	13.39(0.13)	13.44(0.18)	13.87(0.22)	13.77(0.18)	14.75(1.95)	13.70(0.11)	13.71(0.11)
243	264	13.03(0.11)	13.04(0.10)	13.29(0.16)	13.22(0.13)	15.07(1.43)	13.40(0.10)	13.41(0.11)
243	338	13.06(0.13)	13.08(0.12)	13.27(0.17)	13.23(0.16)	15.01(2.02)	13.47(0.09)	13.52(0.10)
243	73	13.38(0.15)	13.37(0.16)	13.60(0.16)	13.53(0.15)	15.15(1.90)	13.76(0.11)	13.74(0.12)
243	458	13.42(0.33)	13.40(0.33)	13.56(0.29)	13.48(0.31)	15.18(2.64)	13.75(0.20)	13.78(0.20)
243	167	13.27(0.15)	13.25(0.14)	13.52(0.16)	13.39(0.14)	15.20(1.59)	13.63(0.11)	13.66(0.10)
243	443	13.52(0.14)	13.47(0.14)	13.63(0.13)	13.60(0.13)	15.04(1.71)	13.86(0.11)	13.86(0.12)
243	208	13.28(0.17)	13.27(0.17)	13.46(0.17)	13.41(0.17)	15.07(1.73)	13.65(0.11)	13.65(0.13)
243	300	13.14(0.15)	13.15(0.14)	13.33(0.17)	13.27(0.14)	15.09(1.15)	13.53(0.12)	13.55(0.12)
338	420	13.41(0.12)	13.55(0.17)	14.09(0.22)	13.94(0.14)	13.74(1.68)	13.70(0.10)	13.69(0.10)
338	264	13.13(0.11)	13.13(0.11)	13.41(0.17)	13.31(0.11)	14.25(2.34)	13.42(0.08)	13.41(0.10)
338	243	13.15(0.13)	13.16(0.14)	13.55(0.19)	13.41(0.15)	13.84(1.35)	13.41(0.10)	13.39(0.10)
338	73	13.41(0.13)	13.42(0.14)	13.76(0.17)	13.64(0.13)	14.11(1.97)	13.74(0.11)	13.72(0.12)
338	458	13.47(0.26)	13.47(0.26)	13.73(0.26)	13.61(0.25)	14.31(2.11)	13.77(0.20)	13.76(0.16)
338	167	13.33(0.13)	13.32(0.13)	13.59(0.18)	13.47(0.14)	14.31(1.92)	13.67(0.11)	13.63(0.11)
338	443	13.55(0.15)	13.53(0.15)	13.76(0.18)	13.67(0.15)	14.29(1.85)	13.86(0.11)	13.87(0.09)
338	208	13.30(0.14)	13.31(0.15)	13.57(0.18)	13.48(0.15)	14.17(1.19)	13.65(0.13)	13.66(0.13)
338	300	13.24(0.13)	13.21(0.14)	13.48(0.16)	13.38(0.14)	14.08(2.59)	13.54(0.11)	13.55(0.10)
73	420	13.50(0.14)	13.58(0.17)	14.11(0.20)	13.96(0.20)	16.32(2.36)	13.71(0.10)	13.72(0.11)
73	264	13.17(0.12)	13.27(0.12)	13.50(0.15)	13.42(0.16)	16.84(2.10)	13.41(0.08)	13.39(0.10)
73	243	13.22(0.15)	13.29(0.15)	13.71(0.20)	13.54(0.17)	16.30(2.32)	13.44(0.09)	13.41(0.10)
73	338	13.20(0.13)	13.29(0.12)	13.54(0.17)	13.46(0.16)	16.67(1.93)	13.50(0.10)	13.50(0.10)
73	458	13.48(0.28)	13.50(0.26)	13.78(0.24)	13.67(0.25)	16.47(2.92)	13.75(0.19)	13.75(0.21)
73	167	13.36(0.13)	13.40(0.13)	13.66(0.18)	13.57(0.15)	16.60(1.91)	13.67(0.10)	13.68(0.10)
73	443	13.56(0.14)	13.57(0.14)	13.80(0.14)	13.73(0.14)	16.51(2.00)	13.86(0.10)	13.85(0.10)
73	208	13.35(0.14)	13.44(0.14)	13.67(0.17)	13.57(0.15)	17.01(2.09)	13.65(0.11)	13.65(0.13)
73	300	13.23(0.15)	13.32(0.13)	13.56(0.15)	13.50(0.14)	16.88(1.77)	13.53(0.12)	13.54(0.12)
458	420	13.64(0.43)	13.74(0.51)	14.47(0.67)	14.27(0.71)	15.40(2.87)	13.70(0.09)	13.70(0.10)
458	264	13.31(0.25)	13.34(0.27)	13.76(0.53)	13.65(0.55)	16.31(3.37)	13.42(0.10)	13.41(0.10)
458	243	13.36(0.32)	13.35(0.37)	13.96(0.59)	13.78(0.63)	15.89(1.96)	13.41(0.08)	13.42(0.11)
458	338	13.31(0.23)	13.34(0.27)	13.75(0.50)	13.65(0.54)	15.66(2.03)	13.49(0.09)	13.48(0.09)
458	73	13.58(0.30)	13.55(0.36)	14.13(0.58)	13.93(0.58)	15.68(2.73)	13.75(0.12)	13.77(0.13)
458	167	13.44(0.24)	13.47(0.23)	13.89(0.49)	13.70(0.52)	15.87(1.70)	13.64(0.12)	13.64(0.10)
458	443	13.64(0.19)	13.63(0.19)	14.04(0.48)	13.91(0.49)	15.82(2.10)	13.85(0.10)	13.88(0.11)
458	208	13.44(0.23)	13.43(0.25)	13.84(0.47)	13.68(0.50)	16.03(2.06)	13.65(0.13)	13.64(0.12)
458	300	13.37(0.21)	13.41(0.24)	13.84(0.50)	13.71(0.51)	15.64(2.34)	13.54(0.13)	13.53(0.12)

Table S9. Benchmark results for pairwise cross-hospital experiments. Medians and standard deviations of the log of *MAE* between projected duration and observed duration on target admissions for different insurance groups using *OTTEHR*, *TCA*, *CA*, *GFK*, *deepJDOT*, *RSD* and *daregram*. The outperformance ratio of *OTTEHR* to *TCA*/*CA*/*GFK*/*RSD*/*daregram* is defined as the percentage decrease in the median *MAE* from *TCA*/*CA*/*GFK*/*RSD*/*daregram* to *OTTEHR*. The outperformance ratio of *OTTEHR* to *deepJDOT* is defined as the percentage decrease in median of the log-transformed of standard deviation of *MAE* from *deepJDOT* to *OTTEHR*.

Source	Target	*OTTEHR*	*TCA*	*CA*	*GFK*	*deepJDOT*	*RSD*	*daregram*
167	420	13.52(0.15)	13.60(0.26)	14.17(0.22)	14.01(0.21)	15.00(2.18)	13.71(0.12)	13.69(0.10)
167	264	13.20(0.11)	13.23(0.11)	13.54(0.15)	13.46(0.13)	15.83(2.32)	13.41(0.10)	13.41(0.10)
167	243	13.22(0.13)	13.23(0.14)	13.67(0.20)	13.53(0.16)	15.35(1.78)	13.43(0.11)	13.45(0.09)
167	338	13.23(0.12)	13.24(0.11)	13.50(0.17)	13.41(0.13)	15.53(2.81)	13.49(0.11)	13.50(0.09)
167	73	13.44(0.13)	13.45(0.14)	13.81(0.17)	13.68(0.15)	15.57(1.45)	13.74(0.11)	13.74(0.11)
167	458	13.47(0.26)	13.48(0.26)	13.78(0.24)	13.64(0.23)	15.58(2.84)	13.79(0.23)	13.79(0.21)
167	443	13.56(0.14)	13.55(0.13)	13.76(0.13)	13.72(0.13)	15.50(2.55)	13.88(0.13)	13.85(0.14)
167	208	13.33(0.16)	13.35(0.16)	13.65(0.19)	13.53(0.17)	15.76(1.76)	13.65(0.11)	13.68(0.12)
167	300	13.31(0.14)	13.31(0.14)	13.57(0.16)	13.51(0.15)	15.65(1.79)	13.53(0.14)	13.53(0.14)
443	420	13.66(0.15)	13.76(0.19)	14.50(0.23)	14.34(0.21)	14.83(1.28)	13.72(0.11)	13.72(0.11)
443	264	13.41(0.12)	13.44(0.14)	13.82(0.18)	13.70(0.16)	15.63(1.77)	13.41(0.10)	13.41(0.11)
443	243	13.47(0.13)	13.49(0.15)	14.00(0.21)	13.83(0.19)	15.75(3.09)	13.41(0.11)	13.42(0.09)
443	338	13.39(0.14)	13.41(0.15)	13.77(0.19)	13.69(0.18)	15.79(1.35)	13.50(0.09)	13.49(0.10)
443	73	13.59(0.14)	13.60(0.16)	14.12(0.22)	13.98(0.20)	15.86(2.41)	13.74(0.12)	13.73(0.12)
443	458	13.63(0.24)	13.65(0.23)	14.01(0.23)	13.89(0.22)	15.55(1.15)	13.78(0.19)	13.79(0.20)
443	167	13.53(0.13)	13.54(0.15)	13.93(0.20)	13.76(0.17)	15.65(1.51)	13.67(0.12)	13.66(0.11)
443	208	13.52(0.13)	13.50(0.13)	13.82(0.16)	13.75(0.15)	15.89(1.23)	13.65(0.12)	13.66(0.11)
443	300	13.45(0.13)	13.43(0.14)	13.79(0.17)	13.70(0.15)	15.71(2.32)	13.54(0.11)	13.53(0.12)
208	420	13.50(0.16)	13.65(0.26)	14.34(0.35)	14.16(0.32)	14.45(3.00)	13.72(0.10)	13.71(0.11)
208	264	13.26(0.16)	13.28(0.15)	13.67(0.27)	13.49(0.22)	14.98(1.64)	13.40(0.10)	13.41(0.10)
208	243	13.27(0.17)	13.29(0.18)	13.82(0.30)	13.60(0.23)	14.87(1.61)	13.44(0.08)	13.41(0.09)
208	338	13.27(0.15)	13.25(0.14)	13.59(0.27)	13.46(0.21)	14.76(2.79)	13.49(0.09)	13.50(0.10)
208	73	13.49(0.15)	13.51(0.17)	14.00(0.31)	13.81(0.24)	14.91(2.60)	13.76(0.12)	13.73(0.12)
208	458	13.53(0.24)	13.51(0.24)	13.89(0.33)	13.73(0.26)	15.10(2.32)	13.77(0.23)	13.77(0.22)
208	167	13.39(0.14)	13.39(0.15)	13.79(0.25)	13.59(0.19)	14.92(1.01)	13.65(0.11)	13.69(0.11)
208	443	13.59(0.13)	13.58(0.14)	13.87(0.22)	13.76(0.18)	14.90(1.73)	13.87(0.12)	13.87(0.10)
208	300	13.35(0.16)	13.30(0.16)	13.65(0.22)	13.54(0.19)	15.01(1.95)	13.55(0.12)	13.54(0.12)
300	420	13.48(0.16)	13.64(0.32)	14.21(0.28)	14.08(0.27)	14.15(1.46)	13.71(0.09)	13.71(0.10)
300	264	13.19(0.13)	13.17(0.18)	13.51(0.23)	13.40(0.20)	14.35(1.58)	13.41(0.10)	13.43(0.09)
300	243	13.21(0.16)	13.25(0.22)	13.64(0.26)	13.53(0.25)	14.15(2.45)	13.41(0.10)	13.43(0.10)
300	338	13.21(0.13)	13.19(0.14)	13.48(0.20)	13.41(0.19)	14.26(2.29)	13.49(0.09)	13.51(0.09)
300	73	13.46(0.15)	13.46(0.26)	13.85(0.21)	13.70(0.20)	14.33(1.92)	13.74(0.12)	13.73(0.12)
300	458	13.46(0.26)	13.46(0.28)	13.75(0.27)	13.64(0.25)	14.40(1.37)	13.76(0.18)	13.76(0.22)
300	167	13.37(0.14)	13.36(0.20)	13.66(0.24)	13.56(0.20)	14.38(1.77)	13.66(0.11)	13.65(0.10)
300	443	13.58(0.14)	13.56(0.17)	13.79(0.16)	13.73(0.15)	14.53(1.26)	13.88(0.12)	13.89(0.12)
300	208	13.33(0.17)	13.32(0.22)	13.62(0.22)	13.52(0.21)	14.49(2.42)	13.64(0.12)	13.67(0.12)
Population Median		13.36(0.24)	13.38(0.26)	13.68(0.37)	13.58(0.35)	15.30(2.37)	13.62(0.19)	13.62(0.19)
Outperformance ratio of *OTTEHR*		N.A.	1.98	27.39	19.75	89.87	22.89	22.90

Table S10. Benchmark results for pairwise cross-hospital experiments. Medians and standard deviations of the log of *RMSE* between projected duration and observed duration on target admissions for different insurance groups using *OTTEHR*, *TCA*, *CA*, *GFK*, *deepJDOT*, *RSD* and *daregram*. The outperformance ratio of *OTTEHR* to *TCA*/*CA*/*GFK*/*RSD*/*daregram* is defined as the percentage decrease in the median *RMSE* from *TCA*/*CA*/*GFK*/*RSD*/*daregram* to *OTTEHR*. The outperformance ratio of *OTTEHR* to *deepJDOT* is defined as the percentage decrease in median of the log-transformed of standard deviation of *RMSE* from *deepJDOT* to *OTTEHR*.

Source	Target	*OTTEHR*	*TCA*	*CA*	*GFK*	*deepJDOT*	*RSD*	*daregram*
420	264	13.46(0.17)	13.43(0.15)	13.54(0.16)	13.52(0.15)	19.02(1.90)	13.70(0.12)	13.74(0.14)
420	243	13.46(0.21)	13.45(0.17)	13.64(0.17)	13.54(0.17)	18.80(1.92)	13.74(0.16)	13.74(0.16)
420	338	13.49(0.16)	13.46(0.15)	13.57(0.14)	13.54(0.14)	18.60(1.72)	13.78(0.12)	13.81(0.13)
420	73	13.88(0.26)	13.79(0.26)	13.91(0.23)	13.85(0.24)	18.64(1.76)	14.14(0.19)	14.13(0.18)
420	458	14.03(0.69)	13.97(0.71)	14.03(0.67)	14.00(0.69)	18.71(1.34)	14.28(0.51)	14.22(0.54)
420	167	13.73(0.22)	13.65(0.22)	13.80(0.20)	13.71(0.21)	18.43(1.33)	14.02(0.17)	14.00(0.16)
420	443	14.08(0.17)	14.01(0.17)	14.04(0.16)	14.03(0.16)	18.87(1.49)	14.29(0.15)	14.27(0.15)
420	208	13.80(0.30)	13.72(0.30)	13.80(0.28)	13.78(0.29)	18.80(1.97)	14.01(0.24)	14.07(0.28)
420	300	13.63(0.26)	13.60(0.25)	13.68(0.23)	13.64(0.24)	18.91(1.80)	13.93(0.22)	13.88(0.19)
264	420	13.87(0.17)	13.95(0.19)	14.31(0.18)	14.17(0.14)	14.11(1.90)	14.00(0.14)	14.05(0.17)
264	243	13.49(0.19)	13.53(0.21)	13.82(0.20)	13.71(0.18)	14.20(1.78)	13.74(0.16)	13.73(0.16)
264	338	13.51(0.17)	13.47(0.17)	13.66(0.18)	13.60(0.17)	14.31(1.29)	13.81(0.13)	13.79(0.12)
264	73	13.92(0.23)	13.90(0.23)	14.11(0.19)	14.01(0.19)	14.49(1.08)	14.15(0.18)	14.14(0.19)
264	458	14.02(0.62)	14.02(0.62)	14.11(0.57)	14.10(0.59)	14.52(2.00)	14.20(0.57)	14.14(0.41)
264	167	13.75(0.20)	13.75(0.20)	13.93(0.19)	13.84(0.18)	14.61(2.87)	14.05(0.15)	14.00(0.16)
264	443	14.09(0.19)	14.06(0.19)	14.16(0.17)	14.12(0.17)	14.45(1.63)	14.28(0.15)	14.29(0.15)
264	208	13.77(0.33)	13.76(0.33)	13.90(0.29)	13.83(0.30)	14.47(1.20)	14.02(0.27)	14.04(0.25)
264	300	13.66(0.26)	13.64(0.27)	13.80(0.22)	13.73(0.23)	14.34(1.92)	13.88(0.19)	13.91(0.20)
243	420	13.83(0.19)	13.85(0.21)	14.19(0.21)	14.09(0.18)	14.81(1.88)	14.05(0.15)	14.05(0.17)
243	264	13.45(0.17)	13.38(0.18)	13.62(0.17)	13.56(0.16)	15.08(1.35)	13.70(0.15)	13.73(0.16)
243	338	13.45(0.18)	13.44(0.17)	13.59(0.18)	13.57(0.17)	15.04(1.97)	13.77(0.13)	13.84(0.14)
243	73	13.94(0.26)	13.90(0.27)	14.07(0.22)	14.00(0.23)	15.17(1.83)	14.14(0.19)	14.12(0.19)
243	458	14.05(0.74)	14.02(0.75)	14.13(0.70)	14.06(0.72)	15.43(2.55)	14.21(0.47)	14.27(0.46)
243	167	13.75(0.20)	13.73(0.20)	13.90(0.18)	13.76(0.19)	15.22(1.52)	14.01(0.18)	14.03(0.15)
243	443	14.07(0.19)	14.04(0.20)	14.10(0.17)	14.08(0.17)	15.12(1.63)	14.27(0.14)	14.31(0.16)
243	208	13.80(0.32)	13.76(0.33)	13.87(0.29)	13.83(0.30)	15.13(1.64)	14.04(0.23)	14.05(0.24)
243	300	13.64(0.30)	13.61(0.30)	13.74(0.26)	13.68(0.27)	15.11(1.07)	13.90(0.21)	13.91(0.21)
338	420	13.82(0.19)	13.95(0.17)	14.40(0.21)	14.25(0.14)	14.15(1.59)	14.04(0.15)	14.04(0.14)
338	264	13.47(0.16)	13.46(0.16)	13.72(0.17)	13.60(0.13)	14.34(2.27)	13.74(0.13)	13.73(0.14)
338	243	13.50(0.19)	13.55(0.19)	13.86(0.20)	13.76(0.17)	14.04(1.22)	13.76(0.15)	13.72(0.14)
338	73	13.89(0.21)	13.93(0.22)	14.16(0.20)	14.03(0.18)	14.46(1.87)	14.13(0.18)	14.11(0.19)
338	458	14.06(0.63)	14.05(0.63)	14.17(0.58)	14.10(0.60)	14.57(2.03)	14.24(0.53)	14.24(0.35)
338	167	13.72(0.20)	13.74(0.19)	13.95(0.20)	13.84(0.18)	14.43(1.81)	14.03(0.17)	14.03(0.15)
338	443	14.07(0.20)	14.05(0.20)	14.18(0.19)	14.12(0.19)	14.51(1.74)	14.27(0.15)	14.31(0.13)
338	208	13.76(0.26)	13.76(0.26)	13.95(0.23)	13.85(0.24)	14.44(1.05)	14.05(0.23)	14.05(0.26)
338	300	13.61(0.26)	13.62(0.27)	13.81(0.23)	13.72(0.24)	14.30(2.50)	13.89(0.20)	13.92(0.21)
73	420	13.91(0.18)	13.94(0.18)	14.39(0.19)	14.24(0.19)	16.32(2.32)	14.05(0.13)	14.06(0.16)
73	264	13.47(0.16)	13.52(0.14)	13.77(0.16)	13.69(0.17)	16.84(2.09)	13.72(0.12)	13.70(0.15)
73	243	13.63(0.21)	13.60(0.19)	13.97(0.20)	13.82(0.18)	16.30(2.31)	13.77(0.15)	13.75(0.14)
73	338	13.49(0.17)	13.52(0.15)	13.79(0.18)	13.72(0.17)	16.67(1.91)	13.80(0.14)	13.79(0.13)
73	458	14.04(0.64)	14.00(0.64)	14.20(0.57)	14.12(0.59)	16.49(2.87)	14.27(0.50)	14.19(0.55)
73	167	13.78(0.19)	13.75(0.19)	13.97(0.19)	13.88(0.18)	16.60(1.89)	14.03(0.14)	14.03(0.16)
73	443	14.06(0.19)	14.03(0.19)	14.18(0.17)	14.15(0.18)	16.51(1.97)	14.30(0.13)	14.26(0.13)
73	208	13.79(0.29)	13.77(0.29)	14.00(0.25)	13.91(0.25)	17.01(2.07)	14.05(0.22)	14.02(0.24)
73	300	13.64(0.27)	13.66(0.25)	13.86(0.20)	13.79(0.22)	16.88(1.74)	13.88(0.22)	13.89(0.22)
458	420	14.07(0.68)	14.10(0.53)	14.73(0.66)	14.54(0.70)	15.41(2.79)	14.06(0.14)	14.04(0.14)
458	264	13.67(0.33)	13.62(0.34)	14.03(0.53)	13.93(0.55)	16.31(3.35)	13.75(0.16)	13.71(0.14)
458	243	13.76(0.53)	13.69(0.51)	14.24(0.59)	14.05(0.64)	15.90(1.92)	13.75(0.12)	13.73(0.16)
458	338	13.65(0.31)	13.59(0.40)	14.04(0.51)	13.93(0.54)	15.67(1.99)	13.80(0.12)	13.78(0.12)
458	73	14.06(0.55)	13.98(0.42)	14.42(0.57)	14.24(0.57)	15.69(2.66)	14.15(0.19)	14.19(0.18)
458	167	13.85(0.41)	13.86(0.31)	14.19(0.48)	14.02(0.52)	15.89(1.65)	13.99(0.16)	14.00(0.14)
458	443	14.15(0.25)	14.12(0.22)	14.37(0.44)	14.28(0.46)	15.83(2.04)	14.27(0.13)	14.30(0.14)
458	208	13.87(0.38)	13.81(0.35)	14.19(0.47)	14.01(0.51)	16.04(1.99)	14.03(0.24)	14.03(0.22)
458	300	13.81(0.31)	13.76(0.33)	14.22(0.49)	14.08(0.51)	15.66(2.29)	13.90(0.23)	13.87(0.22)

Table S11. Benchmark results for pairwise cross-hospital experiments (continued). Medians and standard deviations of the log of *RMSE* between projected duration and observed duration on target admissions for different insurance groups using *OTTEHR*, *TCA*, *CA*, *GFK*, *deepJDOT*, *RSD* and *daregram*. The outperformance ratio of *OTTEHR* to *TCA*/*CA*/*GFK*/*RSD*/*daregram* is defined as the percentage decrease in the median *RMSE* from *TCA*/*CA*/*GFK*/*RSD*/*daregram* to *OTTEHR*. The outperformance ratio of *OTTEHR* to *deepJDOT* is defined as the percentage decrease in median of the log-transformed of standard deviation of *RMSE* from *deepJDOT* to *OTTEHR*.

Source	Target	*OTTEHR*	*TCA*	*CA*	*GFK*	*deepJDOT*	*RSD*	*daregram*
167	420	13.93(0.19)	13.98(0.28)	14.45(0.22)	14.28(0.20)	15.05(2.10)	14.08(0.17)	14.04(0.14)
167	264	13.54(0.14)	13.53(0.18)	13.80(0.16)	13.71(0.14)	15.84(2.27)	13.72(0.14)	13.73(0.14)
167	243	13.57(0.17)	13.54(0.19)	13.97(0.20)	13.83(0.17)	15.36(1.71)	13.77(0.15)	13.77(0.15)
167	338	13.57(0.16)	13.52(0.15)	13.77(0.18)	13.68(0.15)	15.54(2.77)	13.79(0.15)	13.79(0.11)
167	73	13.86(0.20)	13.87(0.21)	14.18(0.18)	14.05(0.19)	15.59(1.38)	14.14(0.19)	14.12(0.21)
167	458	14.01(0.63)	14.00(0.64)	14.17(0.56)	14.08(0.60)	15.77(2.76)	14.28(0.56)	14.28(0.52)
167	443	14.05(0.20)	14.01(0.19)	14.17(0.16)	14.12(0.18)	15.52(2.52)	14.29(0.16)	14.28(0.18)
167	208	13.75(0.30)	13.74(0.30)	13.97(0.26)	13.85(0.27)	15.78(1.72)	14.06(0.23)	14.08(0.25)
167	300	13.72(0.26)	13.67(0.26)	13.89(0.22)	13.81(0.23)	15.65(1.72)	13.89(0.26)	13.88(0.24)
443	420	14.07(0.19)	14.10(0.20)	14.75(0.22)	14.61(0.20)	14.88(1.19)	14.05(0.17)	14.08(0.14)
443	264	13.70(0.15)	13.69(0.16)	14.07(0.17)	13.94(0.16)	15.64(1.73)	13.74(0.16)	13.71(0.16)
443	243	13.80(0.17)	13.79(0.19)	14.25(0.22)	14.08(0.18)	15.75(3.04)	13.75(0.16)	13.76(0.14)
443	338	13.68(0.17)	13.67(0.18)	14.01(0.18)	13.92(0.18)	15.79(1.29)	13.80(0.12)	13.79(0.12)
443	73	13.98(0.19)	14.01(0.21)	14.38(0.21)	14.27(0.20)	15.87(2.35)	14.14(0.21)	14.13(0.21)
443	458	14.12(0.61)	14.13(0.62)	14.38(0.52)	14.27(0.54)	15.77(1.03)	14.27(0.47)	14.28(0.51)
443	167	13.84(0.18)	13.88(0.20)	14.21(0.21)	14.05(0.19)	15.66(1.46)	14.04(0.16)	14.03(0.17)
443	208	13.87(0.26)	13.85(0.26)	14.14(0.21)	14.07(0.22)	15.90(1.15)	14.05(0.22)	14.06(0.23)
443	300	13.76(0.21)	13.74(0.22)	14.06(0.19)	14.00(0.18)	15.72(2.28)	13.89(0.20)	13.89(0.21)
208	420	13.93(0.20)	14.04(0.27)	14.62(0.34)	14.45(0.30)	14.61(2.92)	14.09(0.13)	14.05(0.17)
208	264	13.60(0.21)	13.60(0.20)	13.90(0.27)	13.77(0.22)	15.00(1.55)	13.69(0.15)	13.73(0.15)
208	243	13.64(0.21)	13.63(0.23)	14.12(0.29)	13.91(0.23)	14.89(1.48)	13.77(0.13)	13.74(0.14)
208	338	13.60(0.21)	13.55(0.19)	13.86(0.27)	13.75(0.22)	14.81(2.73)	13.79(0.13)	13.81(0.12)
208	73	13.94(0.22)	13.98(0.22)	14.34(0.31)	14.18(0.25)	15.05(2.52)	14.13(0.21)	14.13(0.18)
208	458	14.07(0.54)	14.07(0.55)	14.30(0.51)	14.18(0.50)	15.25(2.22)	14.27(0.58)	14.27(0.51)
208	167	13.81(0.19)	13.81(0.21)	14.09(0.24)	13.94(0.20)	14.99(0.90)	14.03(0.15)	14.05(0.16)
208	443	14.09(0.17)	14.08(0.19)	14.26(0.20)	14.17(0.18)	15.01(1.65)	14.29(0.15)	14.30(0.12)
208	300	13.76(0.27)	13.67(0.28)	14.01(0.26)	13.89(0.25)	15.03(1.87)	13.90(0.22)	13.91(0.21)
300	420	13.92(0.21)	14.01(0.30)	14.50(0.27)	14.35(0.25)	14.34(1.35)	14.07(0.14)	14.07(0.14)
300	264	13.53(0.18)	13.50(0.24)	13.81(0.23)	13.71(0.21)	14.44(1.49)	13.74(0.15)	13.76(0.15)
300	243	13.58(0.21)	13.61(0.30)	13.95(0.24)	13.83(0.24)	14.29(2.35)	13.75(0.14)	13.74(0.14)
300	338	13.58(0.17)	13.52(0.21)	13.76(0.19)	13.70(0.19)	14.38(2.20)	13.79(0.13)	13.83(0.13)
300	73	13.93(0.23)	13.99(0.28)	14.23(0.21)	14.12(0.21)	14.46(1.84)	14.15(0.20)	14.10(0.19)
300	458	14.08(0.59)	14.09(0.61)	14.20(0.54)	14.16(0.55)	14.73(1.23)	14.24(0.49)	14.24(0.55)
300	167	13.79(0.20)	13.77(0.24)	14.03(0.23)	13.93(0.21)	14.51(1.68)	14.03(0.17)	14.02(0.15)
300	443	14.10(0.17)	14.08(0.20)	14.21(0.17)	14.17(0.17)	14.72(1.14)	14.30(0.16)	14.32(0.15)
300	208	13.76(0.31)	13.75(0.34)	13.97(0.28)	13.88(0.29)	14.68(2.33)	14.02(0.22)	14.08(0.25)
Population Median		13.79(0.38)	13.78(0.38)	14.05(0.42)	13.95(0.41)	15.34(2.28)	13.99(0.31)	13.99(0.31)
Outperformance ratio of *OTTEHR*		N.A.	-1.00	22.90	14.79	83.33	18.13	18.13

5.3 Computation time

Table S12. Average computational time in seconds per experiment for *OTTEHR*, *TCA*, *CA*, *GFK*, *deepJDOT*, *RSD* and *daregram*.

Experiment	*OTTEHR*	*TCA*	*CA*	*GFK*	*deepJDOT*	*RSD*	*daregram*
Insurance	334	418	11387	454	17350	281	430
Age	273	267	8388	261	16694	280	669
Cross-database	211	226	10311	251	15818	277	294
Cross-hospital	573	589	9056	573	16551	749	1131
Average	348	375	9786	385	16603	397	631

REFERENCES

1. Sun B, Feng J, Saenko K. Correlation alignment for unsupervised domain adaptation. Domain adaptation in computer vision applications. 2017:153-71.
2. Pan SJ, Tsang IW, Kwok JT, Yang Q. Domain adaptation via transfer component analysis. IEEE Transactions on Neural Networks. 2010;22(2):199-210.
3. He H, Wu D. Transfer learning for brain–computer interfaces: A Euclidean space data alignment approach. IEEE Transactions on Biomedical Engineering. 2019;67(2):399-410.
4. Gong B, Shi Y, Sha F, Grauman K. Geodesic flow kernel for unsupervised domain adaptation. In: 2012 IEEE Conference on Computer Vision and Pattern Recognition. IEEE; 2012. p. 2066-73.
5. Long M, Wang J, Ding G, Sun J, Yu PS. Transfer feature learning with joint distribution adaptation. In: Proceedings of the IEEE International Conference on Computer Vision; 2013. p. 2200-7.
6. Damodaran BB, Kellenberger B, Flamary R, Tuia D, Courty N. Deepjdot: Deep joint distribution optimal transport for unsupervised domain adaptation. In: Proceedings of the European conference on computer vision (ECCV); 2018. p. 447-63.
7. Wang S, Wang B, Zhang Z, Heidari AA, Chen H. Class-aware sample reweighting optimal transport for multi-source domain adaptation. Neurocomputing. 2023;523:213-23.
8. Long M, Cao Y, Wang J, Jordan M. Learning transferable features with deep adaptation networks. In: International Conference on Machine Learning. PMLR; 2015. p. 97-105.
9. Long M, Zhu H, Wang J, Jordan MI. Deep transfer learning with joint adaptation networks. In: International conference on machine learning. PMLR; 2017. p. 2208-17.
10. Maria Carlucci F, Porzi L, Caputo B, Ricci E, Rota Bulo S. Autodial: Automatic domain alignment layers. In: Proceedings of the IEEE International Conference on Computer Vision; 2017. p. 5067-75.
11. Ganin Y, Lempitsky V. Unsupervised domain adaptation by backpropagation. In: International Conference on Machine Learning. PMLR; 2015. p. 1180-9.
12. Tzeng E, Hoffman J, Darrell T, Saenko K. Simultaneous deep transfer across domains and tasks. In: Proceedings of the IEEE International Conference on Computer Vision; 2015. p. 4068-76.
13. Ganin Y, Ustinova E, Ajakan H, Germain P, Larochelle H, Laviolette F, et al. Domain-adversarial training of neural networks. Journal of machine learning research. 2016;17(59):1-35.
14. Luo Z, Zou Y, Hoffman J, Fei-Fei LF. Label efficient learning of transferable representations acrosss domains and tasks. Advances in Neural Information Processing Systems. 2017;30.
15. Long M, Cao Z, Wang J, Jordan MI. Conditional adversarial domain adaptation. Advances in neural information processing systems. 2018;31.
16. Zhang Y, Liu T, Long M, Jordan M. Bridging theory and algorithm for domain adaptation. In: International Conference on Machine Learning. PMLR; 2019. p. 7404-13.
17. Peng X, Huang Z, Sun X, Saenko K. Domain agnostic learning with disentangled representations. In: International Conference on Machine Learning. PMLR; 2019. p. 5102-12.
18. Chen X, Wang S, Wang J, Long M. Representation Subspace Distance for Domain Adaptation Regression. In: International Conference on Machine Learning; 2021. p. 1749-59.
19. Nejjar I, Wang Q, Fink O. DARE-GRAM: Unsupervised domain adaptation regression by aligning inverse gram matrices. In: Proceedings of the IEEE/CVF Conference on Computer Vision and Pattern Recognition; 2023. p. 11744-54.
