# Supplementary material for: Transport-based transfer learning on Electronic Health Records: application to detection of treatment disparities
Source: J Am Med Inform Assoc. 2025 Sep 3;33(1):15–25. doi: 10.1093/jamia/ocaf134 (PMC12758479; doi:10.1093/jamia/ocaf134)
Supplement: ocaf134_Supplementary_Data [file ocaf134_supplementary_data.zip › JAMIA_OTTEHR 2.zip!/JAMIA_OTTEHR 2/figs/revised/bound_insurance.pdf]

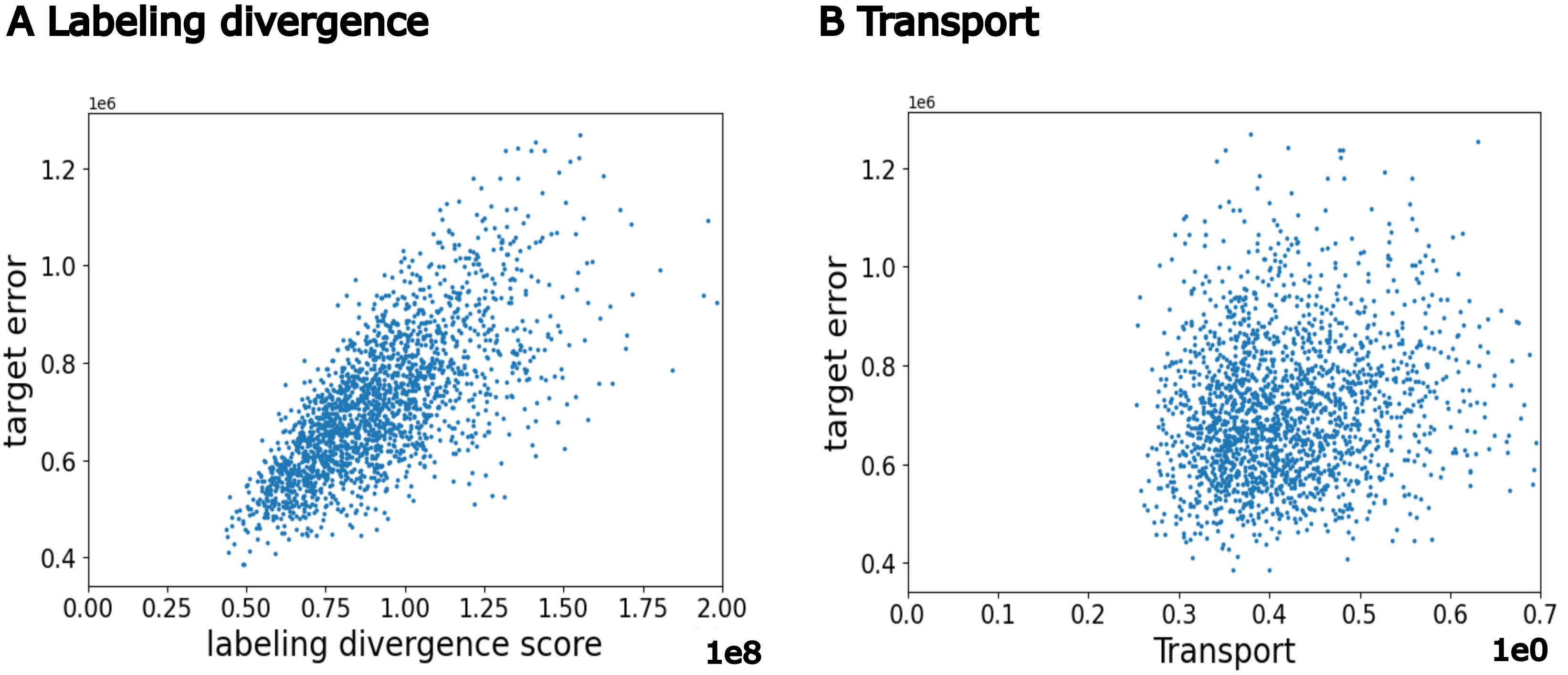

A Labeling divergence
B Transport
1e6
target error
1.2
1.0
0.8
0.6
0.4
0.00
0.25
0.50
0.75
1.00
1.25
1.50
1.75
2.00
labeling divergence score
1e8
1e6
target error
1.2
1.0
0.8
0.6
0.4
0.0
0.1
0.2
0.3
0.4
0.5
0.6
0.7
Transport
1e0
