# Supplementary material for: Transport-based transfer learning on Electronic Health Records: application to detection of treatment disparities
Source: J Am Med Inform Assoc. 2025 Sep 3;33(1):15–25. doi: 10.1093/jamia/ocaf134 (PMC12758479; doi:10.1093/jamia/ocaf134)
Supplement: ocaf134_Supplementary_Data [file ocaf134_supplementary_data.zip › JAMIA_OTTEHR 2.zip!/JAMIA_OTTEHR 2/figs/revised/version_distn_shift.pdf]

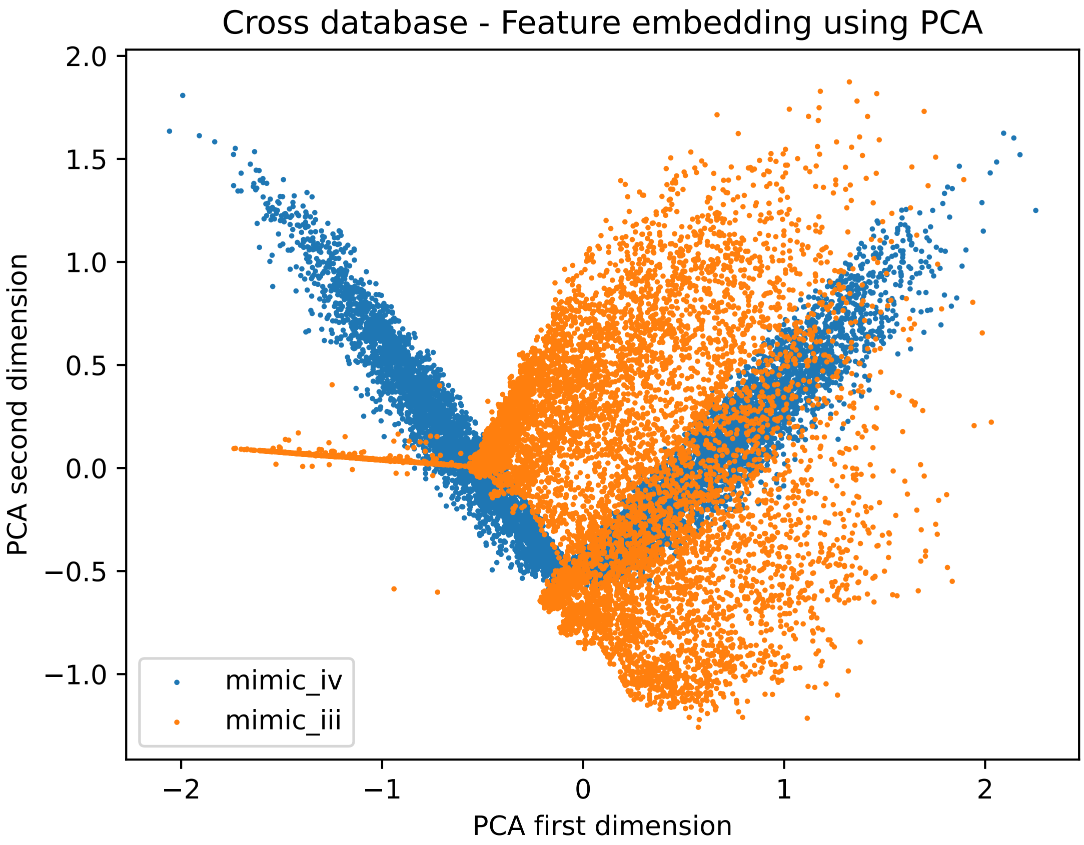
Cross database - Feature embedding using PCA
2.0
1.5
1.0
0.5
0.0
−0.5
−1.0
PCA second dimension
−2
−1
0
1
2
PCA first dimension
mimic_iv
mimic_iii
