# Supplementary material for: Transport-based transfer learning on Electronic Health Records: application to detection of treatment disparities
Source: J Am Med Inform Assoc. 2025 Sep 3;33(1):15–25. doi: 10.1093/jamia/ocaf134 (PMC12758479; doi:10.1093/jamia/ocaf134)
Supplement: ocaf134_Supplementary_Data [file ocaf134_supplementary_data.zip › JAMIA_OTTEHR 2.zip!/JAMIA_OTTEHR 2/figs/revised/accuracy.pdf]

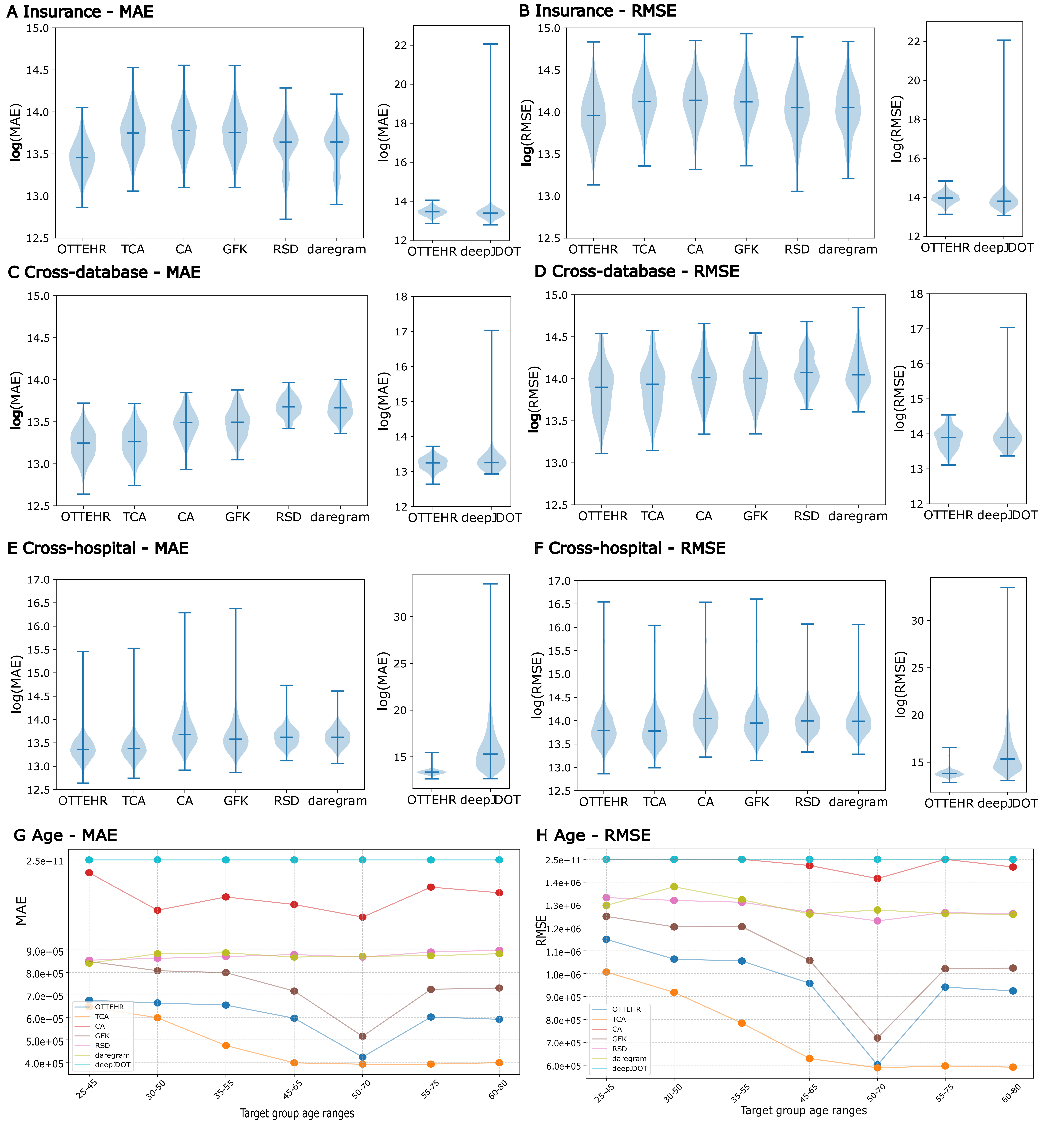
A Insurance - MAE
log(MAE)
15.0
14.5
14.0
13.5
13.0
12.5
OTTEHR
TCA
CA
GFK
RSD
daregram
22
20
18
16
14
12
OTTEHR
deepJDOT
B Insurance - RMSE
log(RMSE)
C Cross-database - MAE
log(MAE)
D Cross-database - RMSE
log(RMSE)
E Cross-hospital - MAE
log(MAE)
F Cross-hospital - RMSE
log(RMSE)
G Age - MAE
MAE
2.5e+11
9.0e+05
8.0e+05
7.0e+05
6.0e+05
5.0e+05
4.0e+05
OTTEHR
TCA
CA
GFK
RSD
daregram
deepJDOT
25-45
30-50
35-55
45-65
50-70
55-75
60-80
Target group age ranges
H Age - RMSE
RMSE
2.5e+11
1.4e+06
1.3e+06
1.2e+06
1.1e+06
1.0e+06
9.0e+05
8.0e+05
7.0e+05
6.0e+05
Target group age ranges
