# Supplementary material for: Transport-based transfer learning on Electronic Health Records: application to detection of treatment disparities
Source: J Am Med Inform Assoc. 2025 Sep 3;33(1):15–25. doi: 10.1093/jamia/ocaf134 (PMC12758479; doi:10.1093/jamia/ocaf134)
Supplement: ocaf134_Supplementary_Data [file ocaf134_supplementary_data.zip › JAMIA_OTTEHR 2.zip!/JAMIA_OTTEHR 2/figs/revised/age_robustness.pdf]

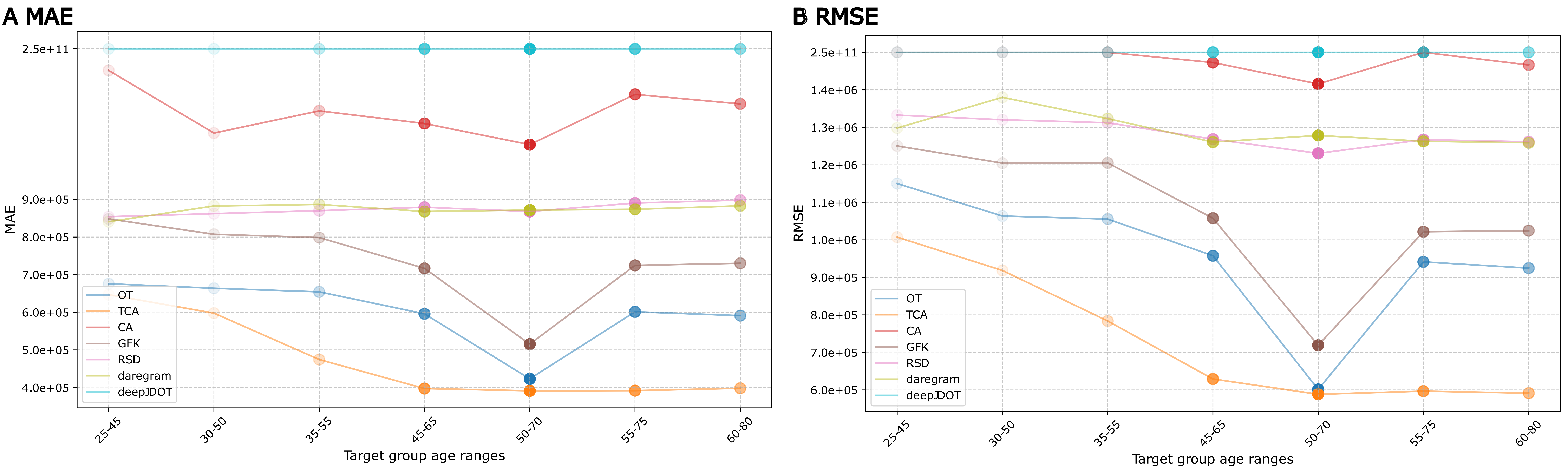
A MAE
B RMSE
MAE
RMSE
2.5e+11
9.0e+05
8.0e+05
7.0e+05
6.0e+05
5.0e+05
4.0e+05
1.4e+06
1.3e+06
1.2e+06
1.1e+06
1.0e+06
25-45
30-50
35-55
45-65
50-70
55-75
60-80
Target group age ranges
OT
TCA
CA
GFK
RSD
daregram
deepJDOT
