# Supplementary material for: Transport-based transfer learning on Electronic Health Records: application to detection of treatment disparities
Source: J Am Med Inform Assoc. 2025 Sep 3;33(1):15–25. doi: 10.1093/jamia/ocaf134 (PMC12758479; doi:10.1093/jamia/ocaf134)
Supplement: ocaf134_Supplementary_Data [file ocaf134_supplementary_data.zip › JAMIA_OTTEHR 2.zip!/JAMIA_OTTEHR 2/figs/revised/OTTEHR-pipeline-framework.pdf]

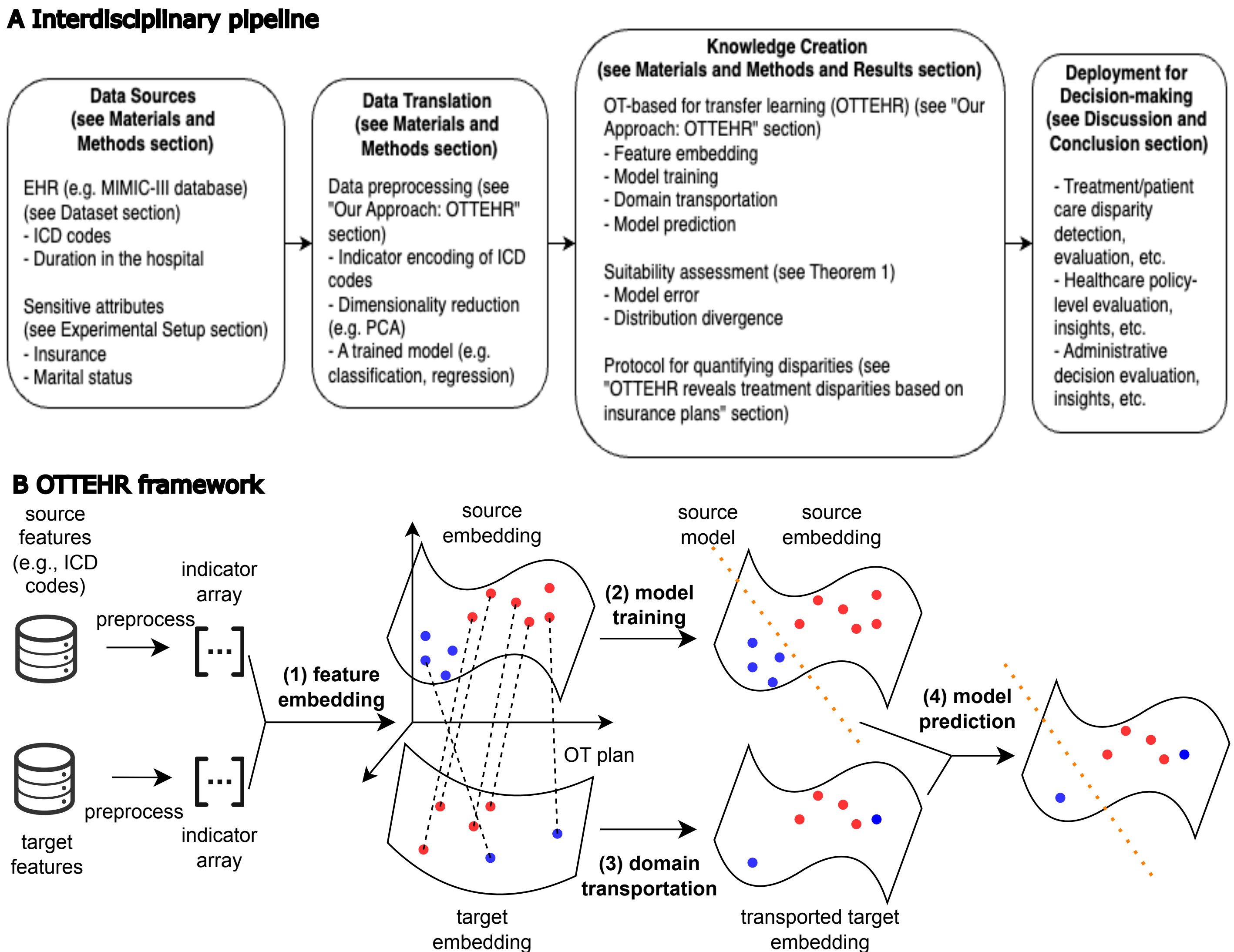
A Interdisciplinary pipeline
Data Sources (see Materials and Methods section)
EHR (e.g. MIMIC-III database) (see Dataset section)
- ICD codes
- Duration in the hospital
Sensitive attributes (see Experimental Setup section)
- Insurance
- Marital status
Data Translation (see Materials and Methods section)
Data preprocessing (see "Our Approach: OTTEHR" section)
- Indicator encoding of ICD codes
- Dimensionality reduction (e.g. PCA)
- A trained model (e.g. classification, regression)
Knowledge Creation (see Materials and Methods and Results section)
OT-based for transfer learning (OTTEHR) (see "Our Approach: OTTEHR" section)
- Feature embedding
- Model training
- Domain transportation
- Model prediction
Suitability assessment (see Theorem 1)
- Model error
- Distribution divergence
Protocol for quantifying disparities (see "OTTEHR reveals treatment disparities based on insurance plans" section)
Deployment for Decision-making (see Discussion and Conclusion section)
- Treatment/patient care disparity detection, evaluation, etc.
- Healthcare policy-level evaluation, insights, etc.
- Administrative decision evaluation, insights, etc.
B OTTEHR framework
source features (e.g., ICD codes)
preprocess
indicator array
target features
preprocess
indicator array
(1) feature embedding
source embedding
OT plan
target embedding
(2) model training
source model
source embedding
(3) domain transportation
transported target embedding
(4) model prediction
