# Supplementary material for: Transport-based transfer learning on Electronic Health Records: application to detection of treatment disparities
Source: J Am Med Inform Assoc. 2025 Sep 3;33(1):15–25. doi: 10.1093/jamia/ocaf134 (PMC12758479; doi:10.1093/jamia/ocaf134)
Supplement: ocaf134_Supplementary_Data [file ocaf134_supplementary_data.zip › JAMIA_OTTEHR 2.zip!/JAMIA_OTTEHR 2/figs/revised/insurance_distn_shift.pdf]

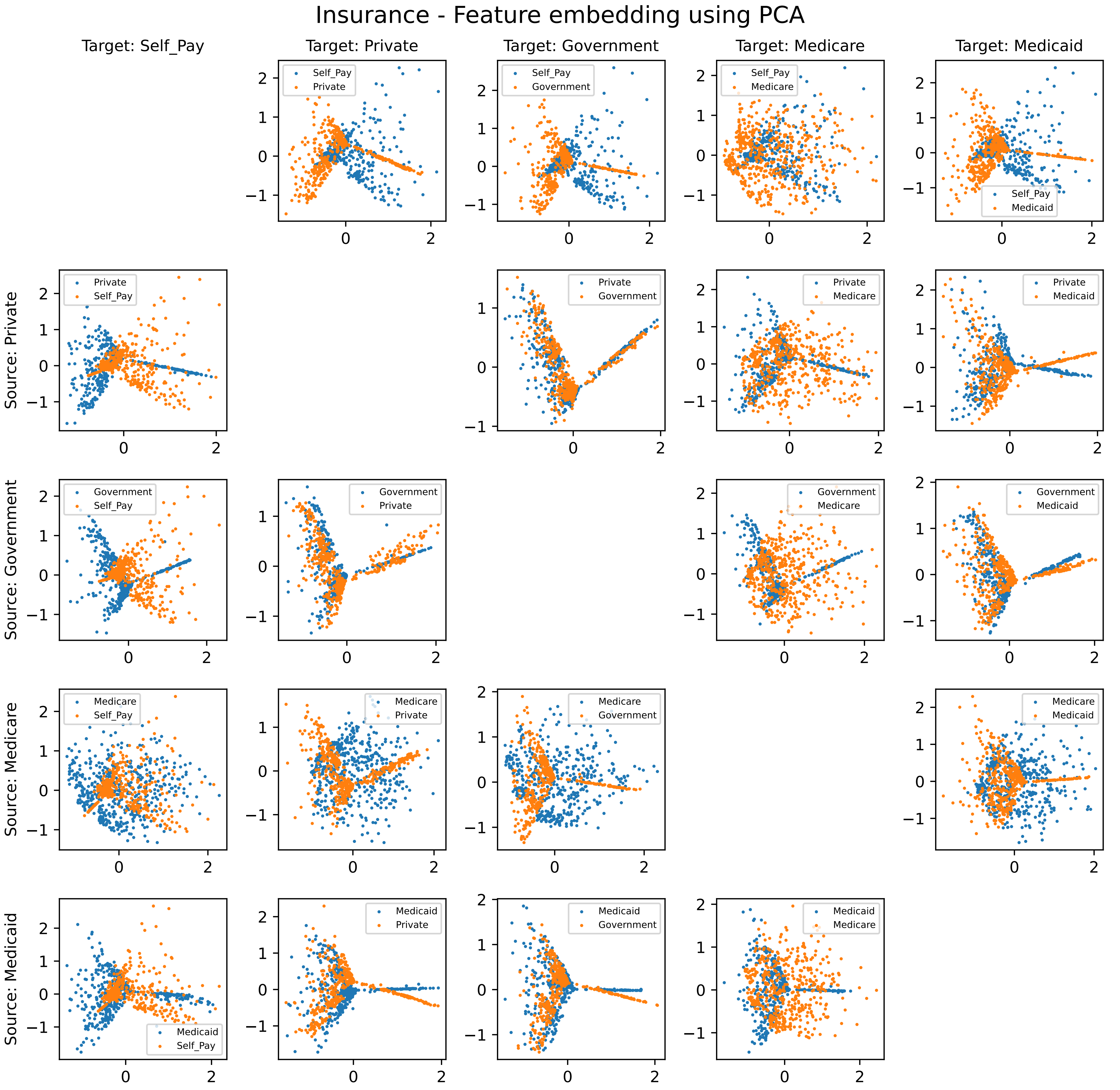
Insurance - Feature embedding using PCA
Target: Self_Pay
Target: Private
Target: Government
Target: Medicare
Target: Medicaid
Source: Private
Source: Government
Source: Medicare
Source: Medicaid
Self_Pay
Private
Government
Medicare
Medicaid
