# Supplementary material for: Transport-based transfer learning on Electronic Health Records: application to detection of treatment disparities
Source: J Am Med Inform Assoc. 2025 Sep 3;33(1):15–25. doi: 10.1093/jamia/ocaf134 (PMC12758479; doi:10.1093/jamia/ocaf134)
Supplement: ocaf134_Supplementary_Data [file ocaf134_supplementary_data.zip › JAMIA_OTTEHR 2.zip!/JAMIA_OTTEHR 2/figs/revised/predicted_observed_coutour_example.pdf]

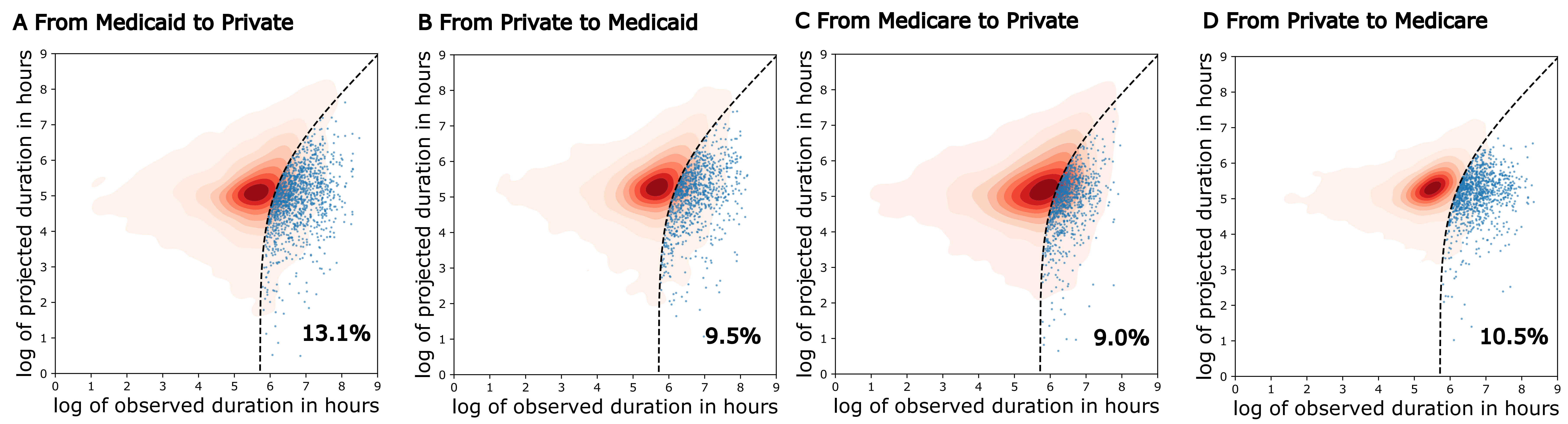

A From Medicaid to Private
B From Private to Medicaid
C From Medicare to Private
D From Private to Medicare
log of projected duration in hours
log of observed duration in hours
0
1
2
3
4
5
6
7
8
9
13.1%
9.5%
9.0%
10.5%
