# Supplementary material for: Transport-based transfer learning on Electronic Health Records: application to detection of treatment disparities
Source: J Am Med Inform Assoc. 2025 Sep 3;33(1):15–25. doi: 10.1093/jamia/ocaf134 (PMC12758479; doi:10.1093/jamia/ocaf134)
Supplement: ocaf134_Supplementary_Data [file ocaf134_supplementary_data.zip › JAMIA_OTTEHR 2.zip!/JAMIA_OTTEHR 2/figs/revised/version_accuracy.pdf]

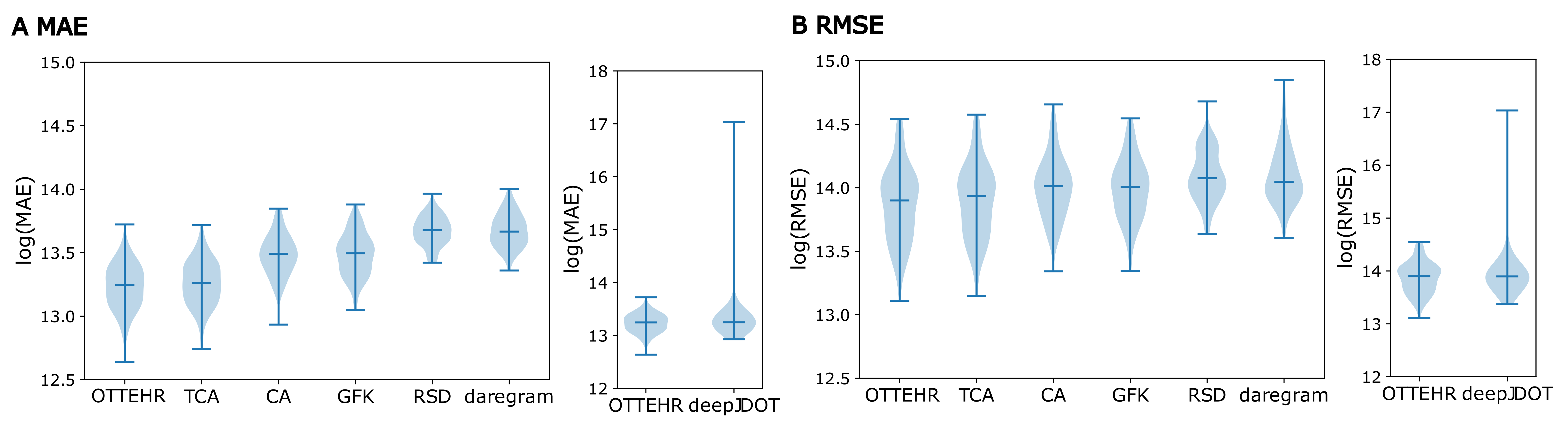

A MAE
B RMSE
log(MAE)
log(RMSE)
15.0
14.5
14.0
13.5
13.0
12.5
18
17
16
15
14
13
12
OTTEHR
TCA
CA
GFK
RSD
daregram
deepJDOT
