# Supplementary material for: Transport-based transfer learning on Electronic Health Records: application to detection of treatment disparities
Source: J Am Med Inform Assoc. 2025 Sep 3;33(1):15–25. doi: 10.1093/jamia/ocaf134 (PMC12758479; doi:10.1093/jamia/ocaf134)
Supplement: ocaf134_Supplementary_Data [file ocaf134_supplementary_data.zip › JAMIA_OTTEHR 2.zip!/JAMIA_OTTEHR 2/figs/mimic/bound_insurance.pdf]

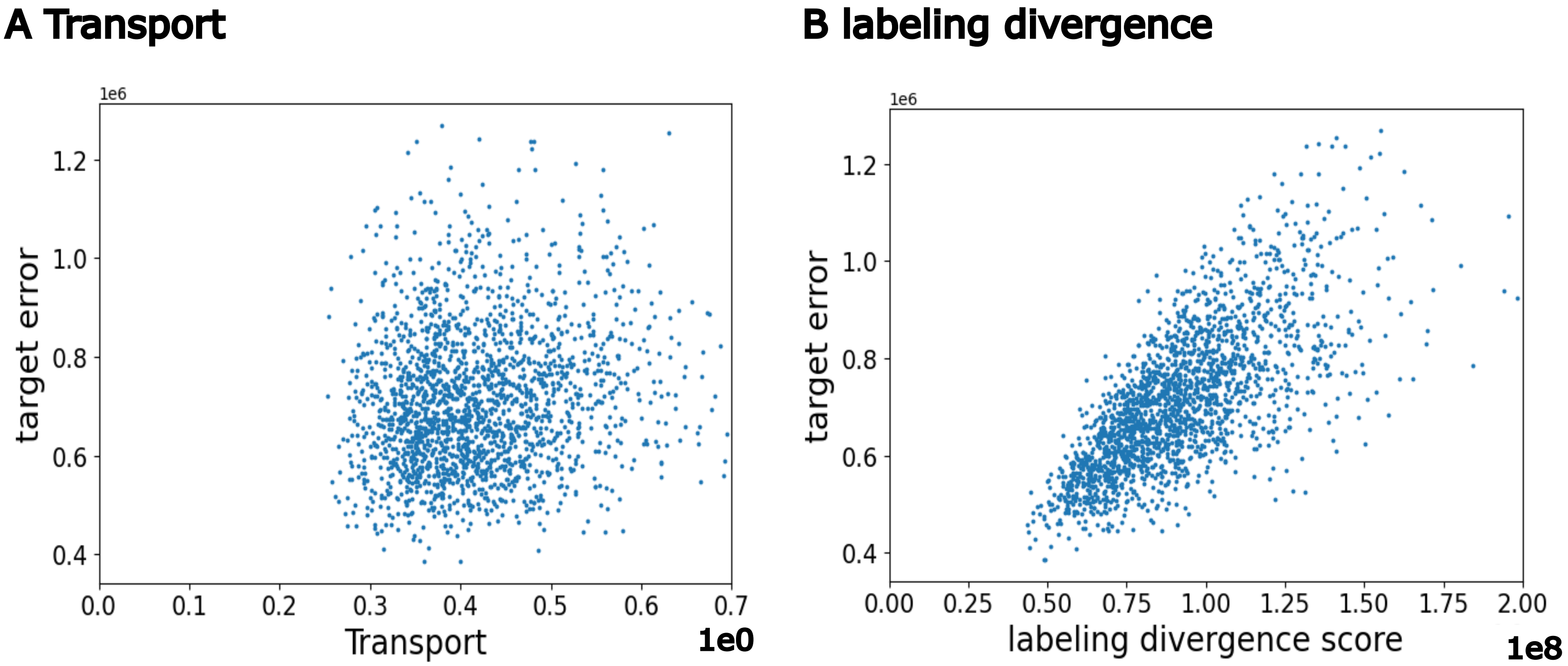
A Transport
1e6
1.2
1.0
0.8
0.6
0.4
target error
0.0
0.1
0.2
0.3
0.4
0.5
0.6
0.7
Transport
1e0
B labeling divergence
1e6
1.2
1.0
0.8
0.6
0.4
target error
0.00
0.25
0.50
0.75
1.00
1.25
1.50
1.75
2.00
labeling divergence score
1e8
