# Supplementary material for: Transport-based transfer learning on Electronic Health Records: application to detection of treatment disparities
Source: J Am Med Inform Assoc. 2025 Sep 3;33(1):15–25. doi: 10.1093/jamia/ocaf134 (PMC12758479; doi:10.1093/jamia/ocaf134)
Supplement: ocaf134_Supplementary_Data [file ocaf134_supplementary_data.zip › JAMIA_OTTEHR 2.zip!/JAMIA_OTTEHR 2/figs/mimic/accuracy_deepJDOT.pdf]

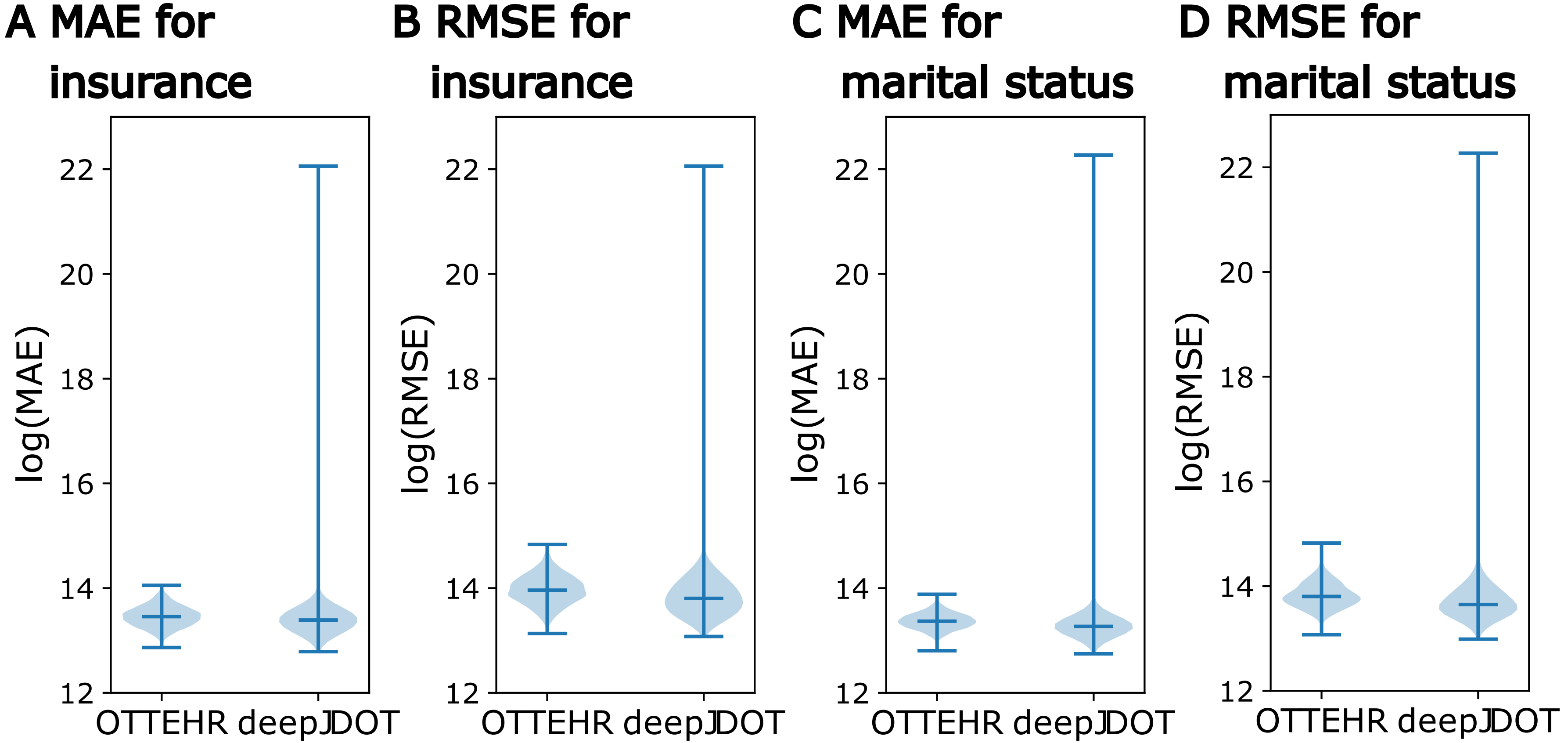
A MAE for insurance
B RMSE for insurance
C MAE for marital status
D RMSE for marital status
log(MAE)
log(RMSE)
12
14
16
18
20
22
OTTEHR
deepJDOT
