# Supplementary material for: Transport-based transfer learning on Electronic Health Records: application to detection of treatment disparities
Source: J Am Med Inform Assoc. 2025 Sep 3;33(1):15–25. doi: 10.1093/jamia/ocaf134 (PMC12758479; doi:10.1093/jamia/ocaf134)
Supplement: ocaf134_Supplementary_Data [file ocaf134_supplementary_data.zip › JAMIA_OTTEHR 2.zip!/JAMIA_OTTEHR 2/figs/mimic/insurance_shorter_predicted_duration_heatmap.pdf]

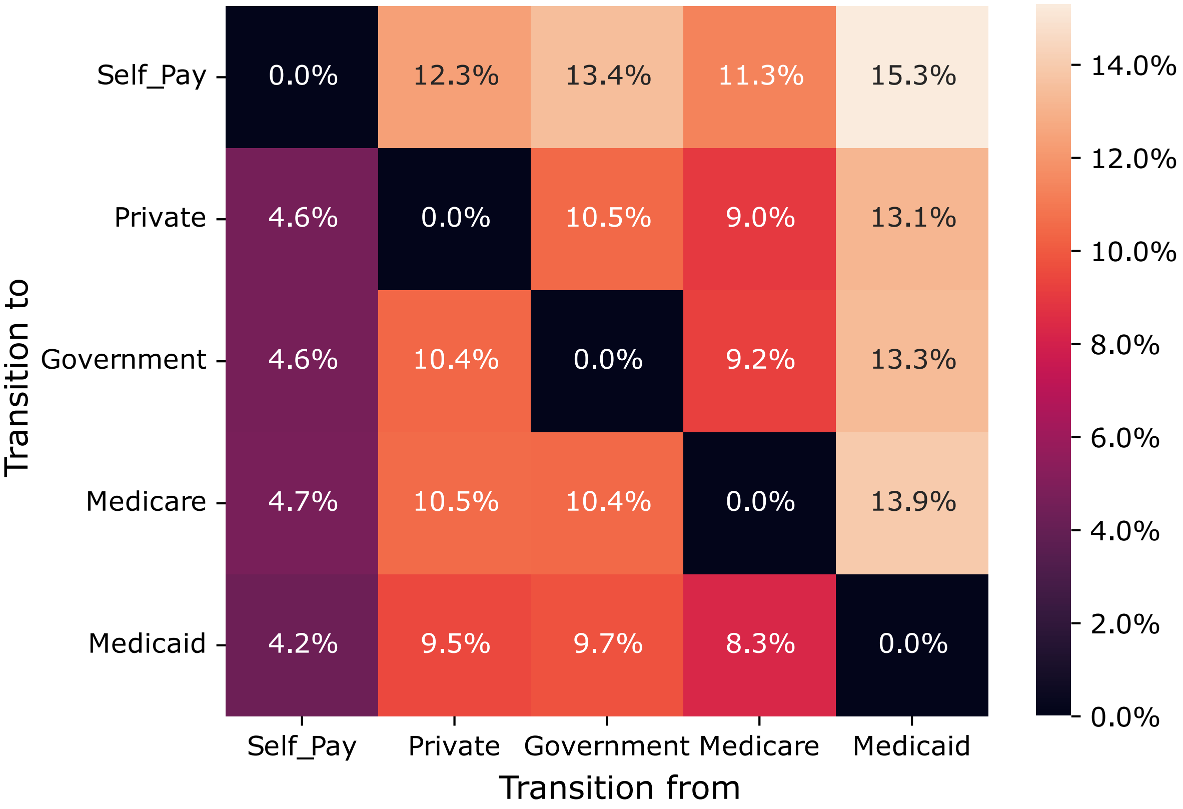

Transition to
Self_Pay
Private
Government
Medicare
Medicaid
0.0%
12.3%
13.4%
11.3%
15.3%
4.6%
0.0%
10.5%
9.0%
13.1%
4.6%
10.4%
0.0%
9.2%
13.3%
4.7%
10.5%
10.4%
0.0%
13.9%
4.2%
9.5%
9.7%
8.3%
0.0%
Self_Pay
Private
Government
Medicare
Medicaid
Transition from
14.0%
12.0%
10.0%
8.0%
6.0%
4.0%
2.0%
0.0%
