# Supplementary material for: Transport-based transfer learning on Electronic Health Records: application to detection of treatment disparities
Source: J Am Med Inform Assoc. 2025 Sep 3;33(1):15–25. doi: 10.1093/jamia/ocaf134 (PMC12758479; doi:10.1093/jamia/ocaf134)
Supplement: ocaf134_Supplementary_Data [file ocaf134_supplementary_data.zip › JAMIA_OTTEHR 2.zip!/JAMIA_OTTEHR 2/figs/mimic/duration_diff.pdf]

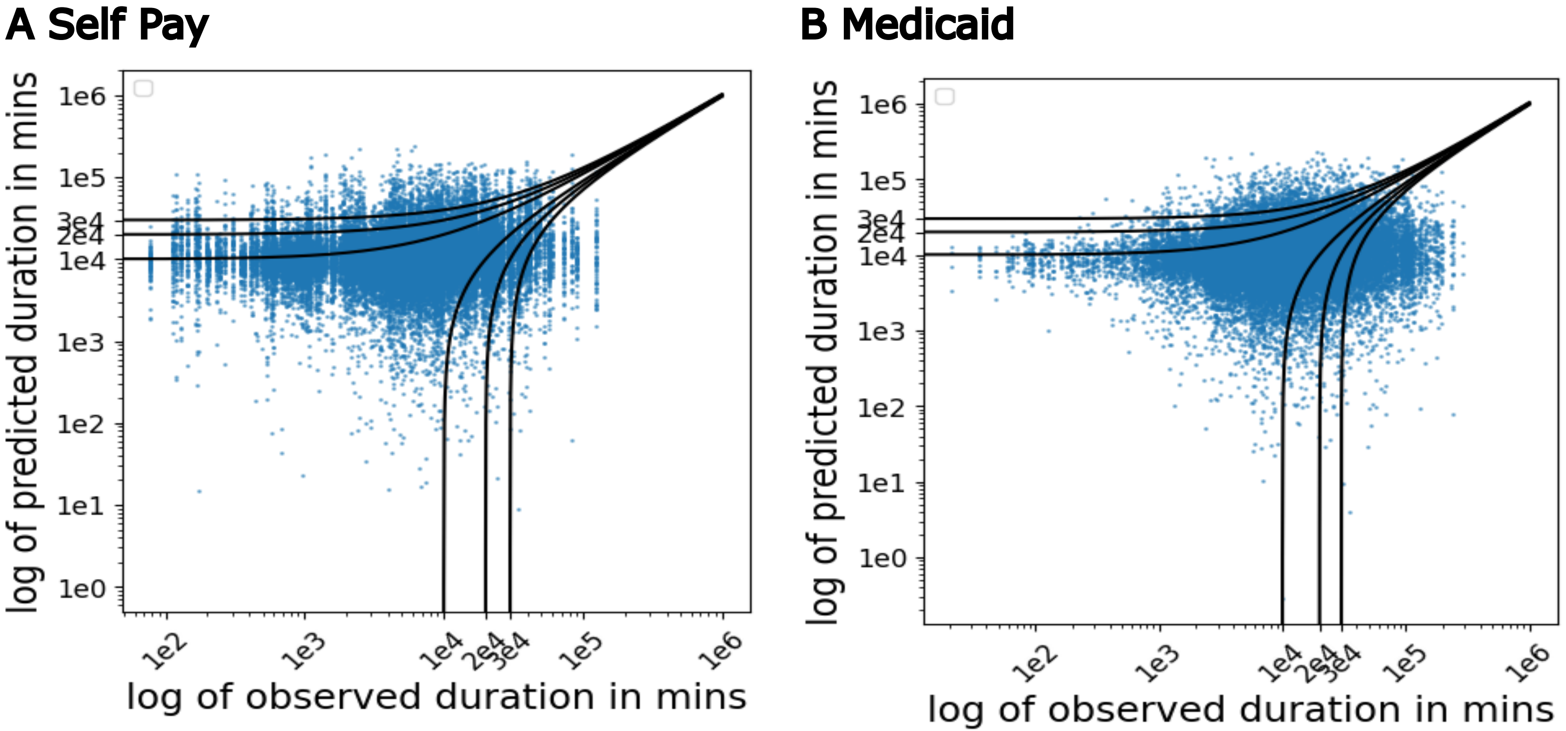

A Self Pay
B Medicaid
log of predicted duration in mins
log of observed duration in mins
1e6
1e5
3e4
2e4
1e4
1e3
1e2
1e1
1e0
