# Supplementary material for: Transport-based transfer learning on Electronic Health Records: application to detection of treatment disparities
Source: J Am Med Inform Assoc. 2025 Sep 3;33(1):15–25. doi: 10.1093/jamia/ocaf134 (PMC12758479; doi:10.1093/jamia/ocaf134)
Supplement: ocaf134_Supplementary_Data [file ocaf134_supplementary_data.zip › JAMIA_OTTEHR 2.zip!/JAMIA_OTTEHR 2/figs/mimic/insurance_longer_predicted_duration_heatmap.pdf]

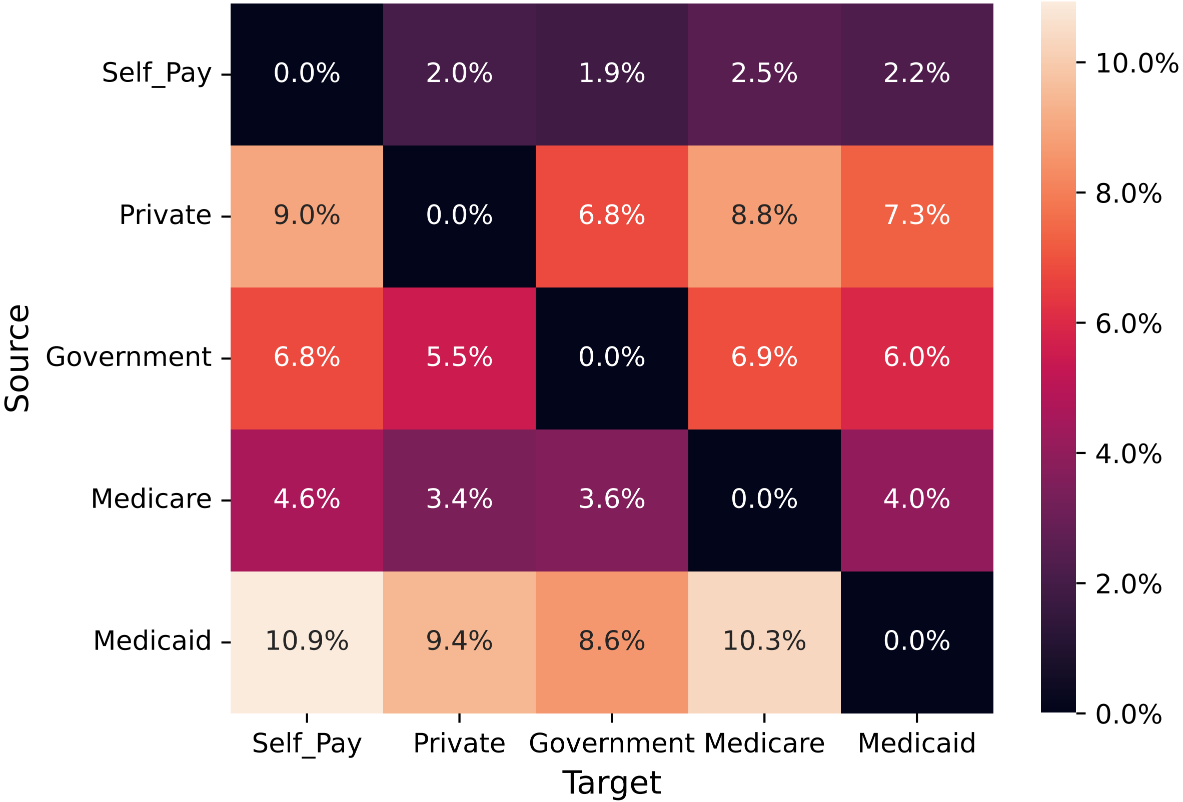
Source
Target
Self_Pay
Private
Government
Medicare
Medicaid
0.0%
2.0%
1.9%
2.5%
2.2%
9.0%
0.0%
6.8%
8.8%
7.3%
6.8%
5.5%
0.0%
6.9%
6.0%
4.6%
3.4%
3.6%
0.0%
4.0%
10.9%
9.4%
8.6%
10.3%
0.0%
10.0%
8.0%
6.0%
4.0%
2.0%
0.0%
